# Human Plastins are Novel Cytoskeletal pH Sensors with a Reduced F-actin Bundling Capacity at Basic pH

**DOI:** 10.1016/j.jmb.2025.169306

**Published:** 2025-06-25

**Authors:** Lucas A. Runyan, Elena Kudryashova, Richa Agrawal, Mubarik Mohamed, Dmitri S. Kudryashov

**Affiliations:** **Department of Chemistry and Biochemistry,** The Ohio State University, Columbus, OH 43210, USA

**Keywords:** plastin, fimbrin, PLS2, LCP1, PLS3, intracellular pH, actin cytoskeleton

## Abstract

Intracellular pH (pH_i_) is a fundamental component of cell homeostasis. Controlled elevations in pH_i_ precede and accompany cell polarization, cytokinesis, and directional migration. pH dysregulation contributes to cancer, neurodegenerative diseases, diabetes, and other metabolic disorders. While cytoskeletal rearrangements are crucial for these processes, only a few cytoskeletal proteins, namely CDC42, cofilin, talin, cortactin, α-actinin, and AIP1 have been documented as pH sensors. Here, we report that actin-bundling proteins plastin 2 (PLS2, aka LCP1) and plastin 3 (PLS3) respond to physiological scale pH fluctuations by a reduced F-actin bundling at alkaline pH. The inhibition of PLS2 actin-bundling activity at elevated pH stems from the reduced affinity of the N-terminal actin-binding domain (ABD1) to actin. In fibroblast cells, elevated cytosolic pH caused the dissociation of ectopically expressed PLS2 and 3 from actin structures, whereas acidic conditions promoted their tighter association with focal adhesions and stress fibers. We identified His207 as one of the pH-sensing residues of PLS2 whose mutation to Lys and Tyr reduces pH sensitivity by enhancing and inhibiting the bundling ability, respectively. Our results suggest that weaker actin bundling by plastin isoforms at alkaline pH favors higher dynamics of the actin cytoskeleton. Therefore, like other cytoskeleton pH sensors, plastins promote disassembly and faster dynamics of cytoskeletal components during cytokinesis and cell migration. Since both plastins are implemented in cancer, their pH sensitivity may contribute to the accelerated proliferation and enhanced invasive and metastatic potentials of cancer cells at alkaline pH_i_.

## Introduction

Intracellular pH (pH_i_) is a fundamental parameter of homeostasis. While in most human cells, pH_i_ is maintained at ~7.2 [1], the exact pH_i_ value varies for different cells and different stages of the cell cycle, raising up to 7.8–8.0 during the transition from G2 to mitosis [2]. pH_i_ homeostasis is frequently distorted under pathological conditions such as cancer, where pH_i_ is constitutively increased [3], and neurodegenerative diseases, where pH_i_ is decreased [4]. pH_i_ is predominantly controlled by Na^+^/H^+^ exchanger 1 (NHE1), which utilizes the Na^+^ gradient to extrude H^+^ into the extracellular space [5]. This process can create short-lived local changes in pH_i_, such as nanodomains with increased pH_i_ (~7.7) at focal adhesions (FAs) essential for their physiological turnover [6-8]. At a larger scale, asymmetric localization and activity of the proton pumps across the cell can produce global pH_i_ gradients essential for cell polarization and directional migration [9-11], with more basic pH_i_ at the protruding cell front, characterized by high levels of actin dynamics, and more acidic pH_i_ at the retracting cell rear [12].

The actin cytoskeleton senses and responds to physiological pH fluctuations through regulatory proteins (*e.g.,* CDC42 [13] and focal adhesion kinase (FAK) [8]), and structural actin-binding proteins (*e.g.,* cofilin [14,15], talin [16], cortactin [17], and actin-interacting protein 1 (AIP1) [18]), whose coordinated activity is essential for facilitating actin dynamics, establishing cell polarity, and promoting migration. At the leading edge, a Rho family GTPase CDC42 is activated by elevated pH_i_ to promote lamellipodial actin assembly [13,19]. Actin recycling is further facilitated by elevated pH_i_ via a release of the inhibitory association of cortactin with cofilin [17], allowing cofilin, whose actin-severing activity is more potent at elevated pH [20-22], to effectively sever actin filaments in lamellipodia [23].

At the adhesion level, elevated pH_i_ promotes the autophosphorylation of FAK, which initiates the recycling of nascent focal adhesions formed at the leading edge, targeting many of them for disassembly [8]. Recycling of mature FAs is also favored by elevated pH_i_ via weakening talin’s affinity for F-actin [16] and promoting cofilin’s severing capacity [20-22], causing disintegration of FAs and disassembly and recycling of actin filaments. It is conceivable that other FA components are also regulated by elevated pH_i_. Thus, it has been shown that α-actinins from *Dictyostelium discoideum* [24] and *Hemicentrotus pulcherrimus* [25] are inhibited by high pH_i_ in the physiological range. However, whether mammalian α-actinin isoforms have a similar pH sensitivity is not known.

**Figure 1.**
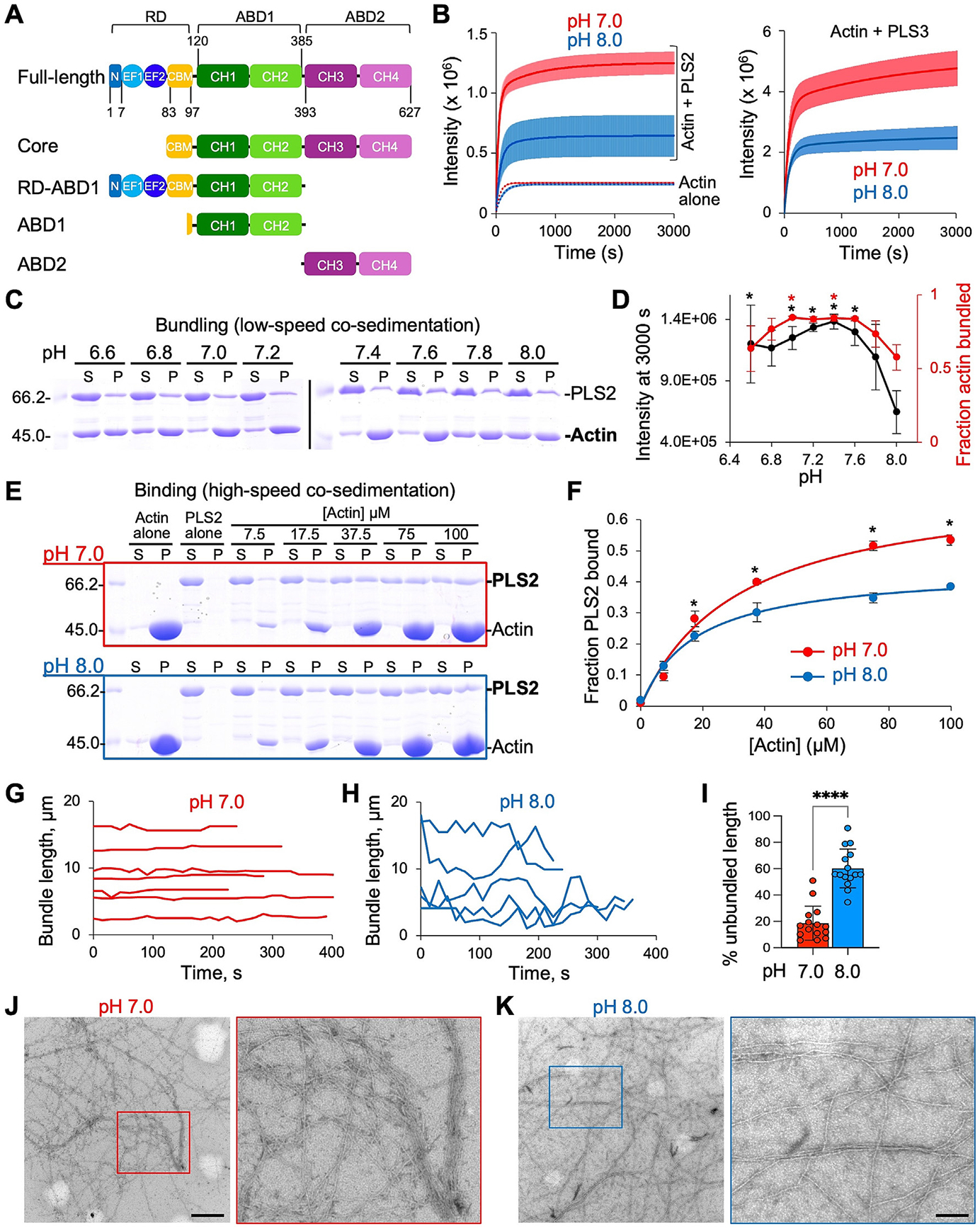
PLS2 and 3 bundling ability is negatively regulated by alkaline pH. (**A**) Schematic of plastin domains and PLS2 constructs used in this study. N,N-terminal 7-a.a. sequence; EF1 and 2, EF-hand motifs; CBM, calmodulin-binding motif; CH1-4, calponin-homology domains; RD, regulatory domain; ABD1 and 2, actin-binding domains. Numbers represent PLS2’s amino acid sequence. (**B**) Light scattering traces of F-actin bundling by PLS2 (left panel; full range of traces at pH from 6.6 to 8.0 is shown in Figure S1A) and that of by PLS3 (right panel) at pH 7 (red) and pH 8 (blue). Dotted lines (left panel) represent light scattering traces upon actin polymerization at pH 7.0 (red) and pH 8.0 (blue); see also Figure S1E. Solid lines represent averaged extrapolated data; colored areas represent SD of the mean (*n* ≥ 3). (**C**) Representative 10% SDS-PAGE gels of supernatant (S) and pellet (P) fractions from low-speed co-sedimentation of actin bundles formed by PLS2 under different buffer pH conditions. (**D**) Light scattering intensity (black) of the reactions at 3000 s (shown in B and in Figure S1A) and the fraction of actin pelleted (red) in low-speed centrifugation assays (shown in C) were plotted as functions of pH. Error bars represent the SD of the mean (*n* ≥ 3); asterisks are color-coded and indicate significant differences from the corresponding value at pH 8.0. *P* values for light-scattering and centrifugation assays are reported in Tables S1 and S2, respectively. (**E**) Representative 10% SDS-PAGE gels of supernatant (S) and pellet (P) fractions from high-speed co-sedimentation of PLS2 with increasing concentrations of F-actin at pH 7.0 and 8.0. (**F**) Quantitation of the high-speed co-sedimentation data (E). Error bars represent SD of the mean (*n* = 3). Asterisks indicate significant difference in fraction of PLS2 bound to actin at 17.5 (*P* = 0.014), 37.5 (*P* = 0.04), 75 (*P* = 0.014), and 100 μM actin (*P* = 0.005). (**G–I**) TIRF microscopy of actin bundling by PLS3. Bundles with lengths visually not increasing over the analyzed period were selected (see Methods); 15 bundles pooled from 3 biological replicates were analyzed. The lengths of individual bundles (or their segments, where all filaments stayed together as a bundle at each time point) were measured and plotted over time for pH 7.0 (**G**) and pH 8.0 (**H**). Representative plots for only some of the analyzed bundles are shown in G and H for clarity; note overall greater variability of the individual bundle lengths, which appear as zigzag instead of straight lines, and frequent unzipping (unbundling) events evidenced by the drops of bundle lengths at pH 8.0, as compared to pH 7.0. (**I**) The difference between maximum and minimum bundle length (percent unbundled) during 2-min evaluation period was expressed as percent of the maximum bundle length for each analyzed bundle; data are presented as mean ± SD, dots represent individual bundles, *n* = 15; ****, *P* < 0.0001 determined by two-tailed *t*-test. (**J, K**) TEM images of bundles formed by PLS2 at pH 7.0 (J) and 8.0 (K). Boxed areas are enlarged for clarity. Scale bars are 500 nm.

Plastin is an actin-bundling protein enriched at the leading edge and in FAs. Like α-actinins, plastins belong to a large family of tandem calponin-homology (*t*-CH) domain actin organizers [26]. Plastins (Figure 1A) contain an N-terminal calcium-binding regulatory domain (RD), followed by two actin-binding domains (ABD1 and ABD2), each composed of two calponin-homology (CH) domains [27]. In the absence of filamentous actin (F-actin), plastins are arranged in a compact horse-shoe-like arrangement [28]. In this form, the ABDs are engaged in an inhibitory association, which permits weak binding to F-actin via one of the domains but does not permit bundling [27,29,30]. Upon binding to F-actin, plastins undergo a structural rearrangement that separates the ABDs, allowing them to bridge actin filaments, cross-linking them into bundles and networks [29,30]. These assemblies are components of cellular structures such as filopodia, lamellipodia, FAs, podosomes, and the cortical cytoskeleton [31,32]. Of the three human plastin isoforms, PLS1 (I-plastin, fimbrin) is expressed in the inner ear stereocilia and intestinal microvilli [33-36], PLS2 (L-plastin, LCP1) in immune cells [33,34,37], and PLS3 (T-plastin) in all solid tissues [33,34,38]. Importantly, PLS2 is abnormally expressed in many cancers, and its expression correlates with enhanced invasive and metastatic potential [39-41], in agreement with its localization to invadopodia and the leading edge [42]. PLS3 contributes to carcinogenesis by fostering drug resistance [43], an activity which has also been suggested for PLS2 in myelomas [44]. PLS3 has also been implicated in promoting migration, invasiveness, and proliferation of cancer cells [45,46].

The actin-bundling activity of plastins is inhibited by Ca^2+^ and enhanced by phosphorylation [27,29,47]. The inhibition by Ca^2+^ is well known, albeit its mechanism remains obscure, as the location of the regulatory, Ca^2+^-binding domain (RD) relative to the actin-binding core is not structurally resolved and only indirectly implied at the ABD1-ABD2 interface [48]. Upon initial binding of two Ca^2+^ ions, the EF-hands in the RD interact with a linker region containing a calmodulin-binding motif (CBM), reorganizing it into an α-helix [27,49]. The RD inhibits F-actin binding by one of the ABDs, whose identity is debated [27,29,30]. The phosphorylation-dependent regulation mechanisms of PLS2 are diverse [42,47,50-55]. Phosphorylation of Ser5 stimulates F-actin bundling in cells [42,47] but a phosphomimetic mutation of PLS2, S5D, does not appear to change its behavior *in vitro* [27], suggesting the possible participation of unknown cellular partners. Identified as a common phosphorylation site in high-throughput studies [50-54], Ser406 is strategically positioned to control the inhibitory association between ABDs. The S406E phosphomimetic mutation weakens binding between ABD1 and ABD2, releasing the autoinhibition and leading to dramatically potentiated bundling both *in cellulo* and *in vitro*, even in the presence of Ca^2+^ [29].

In this study, we demonstrate that F-actin bundling by PLS2 and PLS3 is pH-dependent, with weakened bundling activity at basic pH. We found that PLS2’s pH-sensitivity stems from the inhibited F-actin-binding ability of ABD1 at basic pH, while actin binding by ABD2 was less affected by pH. We validated the physiological importance of pH-sensing by PLS2 through two independent methods. First, by changing the pH_i_ of fibroblasts, we found that PLS2 redistributed from mainly F-actin-associated to diffuse cytosolic upon transition from neutral to basic pH. Second, we generated mutants of PLS2 whose actin-bundling activity was pH-independent. These mutants allowed us to show the cellular consequences of the loss of pH-sensitivity of PLS2, thereby proving that pH-sensitivity is a *bona fide* sensory mode of PLS2, with physiologically relevant consequences to its activity and distribution in cells. This study suggests that the pH sensitivity of PLS2 and PLS3 likely contributes to the reported enhanced migration, invasion, and proliferation of cancer cells expressing these plastin isoforms.

## Results

### Human plastin isoforms PLS2 and PLS3 bundle F-actin in a pH-dependent manner

We evaluated the pH sensitivity of F-actin bundling by PLS2 and PLS3 via light scattering (Figure 1B) and by PLS2 via differential centrifugation under conditions sufficient to pellet bundled F-actin but not individual filaments (20,000*g*, 20 min; Figure 1C, D). F-actin was bundled by PLS2 across all conditions tested, from pH 6.6 to pH 8.0, with the most intense bundling occurring between pH 7.0 and 7.6 as judged from both higher intensity of light scattering and more actin in the pellet at low-speed centrifugation (Figure 1B-D; S1A; Tables S1, S2). At pH 7.8 and 8.0, F-actin bundling by PLS2 was progressively decreased (Figure 1D). Similarly, PLS3 bundled F-actin at pH 7.0 more efficiently than at pH 8.0 (Figure 1B, right panel). These data suggest that PLS2 and PLS3 bundle F-actin in a pH-dependent manner.

PLS2’s binding to F-actin was measured at pH 7.0 and 8.0 via high-speed co-sedimentation (300,000*g*, 30 min), where the centrifugation speed is sufficient to co-pellet all F-actin together with bound proteins. PLS2 co-sedimented with F-actin significantly more effectively at pH 7.0 than at pH 8.0 (Figure 1E, F), in good agreement with light scattering and low-speed co-sedimentation bundling data (Figure 1B-D).

Examination of PLS3-induced actin bundling by total internal reflection fluorescence (TIRF) microscopy indicated overall similar bundle morphology at both pH conditions, with parallel, anti-parallel, and mixed filament orientation (Videos S1-S3, only pH 7.0 is shown), in accordance with the previous reports [30,56]. However, bundles formed by PLS3 at pH 8.0 appeared less stable and more dynamic, as they underwent frequent unzipping and rearranging, compared to notably less dynamic bundles at pH 7.0 (Figure 1G-I; Videos S4 and S5). Likewise, compared to neutral pH condition, there were visually fewer bundles formed by PLS2 at pH 8.0 and they appeared thinner and less dense by transmission electron microscopy (TEM; Figure 1J, K).

F-actin bundling by plastin is sensitive to ionic strength [57]. Since the same 10 mM HEPES buffer was used to create buffers with different pHs, the increased concentration of NaOH added to the pH 8.0 buffer contributed additional ionic strength equivalent to an extra 6 mM KCl (Supplementary Methods) [58]. To ensure that the inhibited actin bundling behavior of plastin at pH 8.0 is not an artifact of the increased ionic strength, F-actin bundling by PLS2 was re-assessed at pH 7.0 with 36 mM KCl (up from 30 mM KCl) and at pH 8.0 with 24 mM KCl (down from 30 mM KCl). We found that the 6 mM difference in KCl concentration had a negligible effect on actin bundling by PLS2, as reported by light scattering intensity and actin pelleting in low-speed co-sedimentation assays (Figure S1B-D).

Another potential artifact could arise from the pH sensitivity of actin polymerization *per se*, with acidic pH promoting polymerization and basic pH inhibiting it [59,60]. To rule out the possibility that low light scattering intensity reflects the differences in polymerization rather than bundling, we compared the light scattering intensity of F-actin polymerization at pH 7.0 and 8.0. While the actin polymerization rate was slightly slower at pH 8.0 compared to pH 7.0 (Figure 1B, actin alone; S1E), the final light scattering intensity for each pH condition was nearly identical (Figure S1E), and the extent of F-actin pelleted at high-speed centrifugation was the same for both pH conditions (Figure S1F, G). These observations agree with the reported minor differences in the critical concentration of F-actin between pH 7.0 and 8.0 [59]. Light scattering experiments using actin filaments pre-polymerized under identical conditions before dilution with a buffer at a specific pH value and the addition of PLS3 corroborated the above findings (Figure S1H). Therefore, the pH dependence of actin polymerization is not the source of decreased actin-bundling activity of plastins at elevated pH.

The 7.8–8.0 pH range, required for inhibition of F-actin bundling by PLS2 (Figure 1D), is at the upper border of the physiological range, as these values are reached during the G2/M transition of the cell cycle [2] and the local pH_i_ near FAs [8] supporting the role of plastins as a physiologically relevant pH-dependent actin regulators.

### The PLS2 actin-binding core retains pH sensitivity

**Figure 2.**
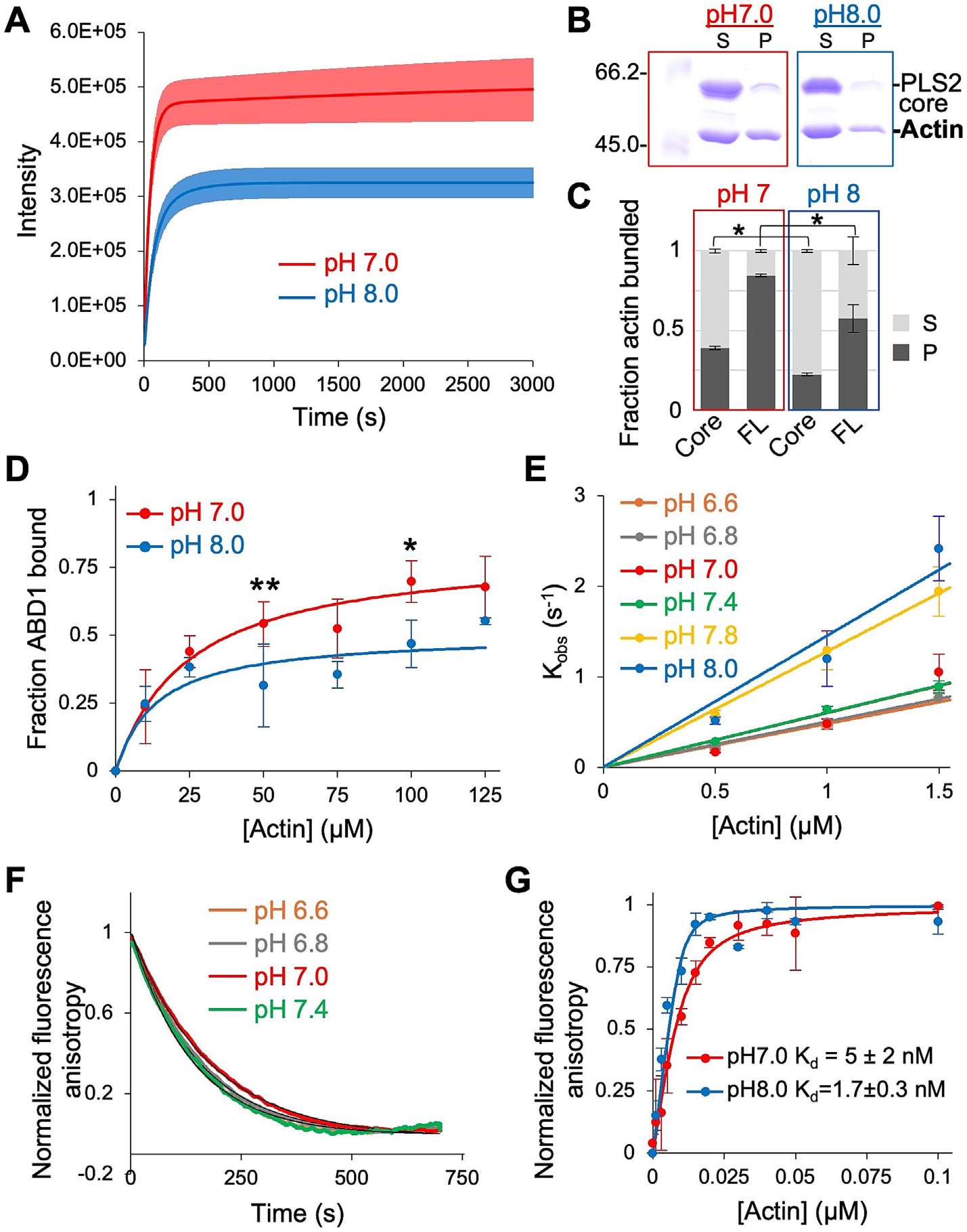
pH-dependence of PLS2 stems from weakened ABD1 binding to actin at basic pH. (**A**) Light scattering traces of F-actin bundling by PLS2-core at pH 7.0 (red) and 8.0 (blue). Solid lines represent averaged extrapolated data; colored areas represent SD of the mean (*n* = 3). (**B**) Representative 10% SDS-PAGE gels of supernatant (S) and pellet (P) fractions from low-speed centrifugation of actin bundles formed by PLS2-core at pH 7.0 and 8.0. (**C**) Quantitation of the low-speed centrifugation data; error bars represent SD of the mean (*n* =3; *, *P* = 0.01). (**D**) Fraction of ABD1 depleted from the supernatant during high-speed co-sedimentation with increasing F-actin concentrations at pH 7.0 (red) and pH 8.0 (blue). Error bars represent SD of the mean (*n* = 3, except for 50 and 100 μM Actin points, where *n* = 6; **, *P* = 0.002; *, *P* = 0.02). (**E**) Slopes from linear fits of $k_{obs}$ values (symbols) determined from kinetic association experiments of FM-ABD2 with F-actin were used to obtain $k_{on}$ values for each pH value (Table 1); error bars represent SD of the mean (*n* ≥ 8). (**F**) Dissociation kinetics (color curves) of FM-ABD2 from F-actin upon competition with excess unlabeled ABD2 were averaged (*n* ≥ 4) and fit to single exponential (black curves) to obtain $k_{off}$ values (Table 1). (**G**) Equilibrium binding data (symbols) of FM-ABD2 binding to phalloidin-stabilized F-actin at pH 7 (red) and pH 8 (blue) fit to a quadratic isotherm (lines). Error bars represent the SD of the mean (*n* = 3). $K_{d}$ values are the mean ± SD of three technical replicates.

The role of individual plastin domains in the observed pH sensitivity of actin bundling was evaluated using PLS2 truncation constructs (Figure 1A). We mainly focused on the characterization of PLS2 in the subsequent experiments, for two reasons: (1) both plastin isoforms showed similar pH dependence; (2) ABD2 of PLS2, when expressed as a separate construct, is soluble and stable, while that of PLS3 is unstable and cannot be purified [27]. A possible involvement of the regulatory calcium-binding domain was tested using PLS2-core (a.a. 83–627), a construct containing both ABDs, but missing the Ca^2+^-binding EF-hands. We chose to include residues N-terminal to CH1 in the construct, as the analogous residues are implicated in actin binding in other members of the *t*-CH protein superfamily [61,62]. PLS2-core bundled F-actin weaker than the full-length (FL) protein, with lower maximum light scattering intensity and less actin pelleted under both pH conditions tested (Figures 2A-C; 1B-D). Yet, basic pH inhibited PLS2-core’s F-actin bundling ability by a factor of ~2 (Figure 2A-C), suggesting that the PLS2-core construct missing the RD is still sensitive to pH, similar to the FL PLS2.

### pH-dependence of PLS2 stems from weakened ABD1 binding to actin at basic pH

While RD contributes to the association between ABD1 and ABD2, its contribution to the binding of ABD1 to actin is insubstantial [27]. Furthermore, RD-ABD1 (a.a. 1–385) has a similar molecular weight to actin, which complicates the SDS-PAGE analysis. Therefore, we used ABD1 (a.a. 120–385), which binds F-actin with an affinity similar to that of RD-ABD1 but migrates on SDS-PAGE substantially faster [27]. In high-speed co-sedimentation assays, consistently less ABD1 co-pelleted with F-actin at pH 8.0, as compared to pH 7.0 at all tested actin concentrations (Figure 2D). Since the statistical significance in triplicates was borderline, we conducted another round of triplicates at actin concentrations 50 and 100 μM. Data combined from the two experiments for these two actin concentrations indeed reached the significance level (Figure 2D). Note that this inhibition at basic pH is consistent with the inhibited interactions of FL PLS2 and PLS2-core with F-actin.

We noticed that only a fraction of ABD1 was found in the pellet (*i.e.*, bound to F-actin) after high-speed co-sedimentation, despite the large excess of actin and apparent saturation of the binding curves at both pH conditions (Figure 2D). This behavior was similar to that of FL PLS2 (Figure 1E, F), except that the latter, but not the former, could also result from actin bundling by the FL protein, which is more difficult to interpret. We hypothesized that the apparent saturation at low binding levels of ABD1 may reflect the limitation of the co-pelleting approach, which does not truly report the binding equilibrium. Indeed, the duration of pelleting required to separate the F-actin complexes from free proteins upon centrifugation is sufficient for the dissociation of weakly bound complexes. Therefore, the method may not adequately report the dissociation constants ($K_{d}$), particularly for reactions characterized by fast dissociation rate constants ($k_{off}$). We confirmed that the incomplete binding is not due to the protein’s structural or functional inadequacy by re-pelleting the supernatant with more added F-actin and demonstrating a similar distribution of ABD1 between pellet (bound to F-actin) and supernatant (unbound) fractions as in the original experiment (Figure S1I).

We next evaluated the pH sensitivity of ABD2 binding to actin. Since ABD2 binds to F-actin with nanomolar $K_{d}$ [29], we took advantage of the sensitivity of fluorescence anisotropy to analyze the pre-steady state binding and dissociation kinetics of fluorescein-maleimide labeled ABD2 (FM-ABD2) using a stopped-flow fluorometer. Interestingly, while the $k_{on}$ values of FM-ABD2 binding to F-actin changed little in the pH 6.6–7.4 range, they increased 2- to 3-fold upon transition to pH 7.8 and 8.0 (Figure 2E; Table 1). The $k_{off}$ values also remained nearly constant in the 6.6–7.4 range of pH (Figure 2F), resulting in $K_{d}$ values fluctuating unsubstantially in the 10–15 nM range (Table 1). We could not measure $k_{off}$ at pH 7.8 and 8.0 due to the low signal-to-noise ratio for reasons we do not fully understand, rendering the data uninterpretable. To address this shortage, the affinity of FM-ABD2 to F-actin was measured independently in equilibrium anisotropy experiments at pH 7.0 and 8.0 (Figure 2G) with phalloidin-stabilized actin to prevent its depolymerization at concentrations below critical. The $K_{d}$ of FM-ABD2 for phalloidin-stabilized F-actin at pH 7.0 was 5 ± 2 nM, *i.e.*, within a reasonable margin of 9.5 nM measured in the kinetic assay [63]. At pH 8.0, the $K_{d}$ of FM-ABD2 binding to F-actin was 1.7 ± 0.3 nM, *i.e.*, ~3-fold stronger than at pH 7.0. Therefore, this difference is mainly accounted for by the higher $k_{on}$ at basic pH, implying that the $k_{off}$ of ABD2 from actin is not substantially affected in the pH 6.6–8.0 range. To summarize, ABD2 binds F-actin stronger at basic pH, in contrast to the behavior of both the FL protein and ABD1, supporting our previous observations that the initial interaction of plastin with actin, which promotes conformational changes required for bundling, is mediated via ABD1 [29].

### Inhibitory ABD1-ABD2 association is not responsible for the weaker bundling ability of PLS2 at basic pH

**Figure 3.**
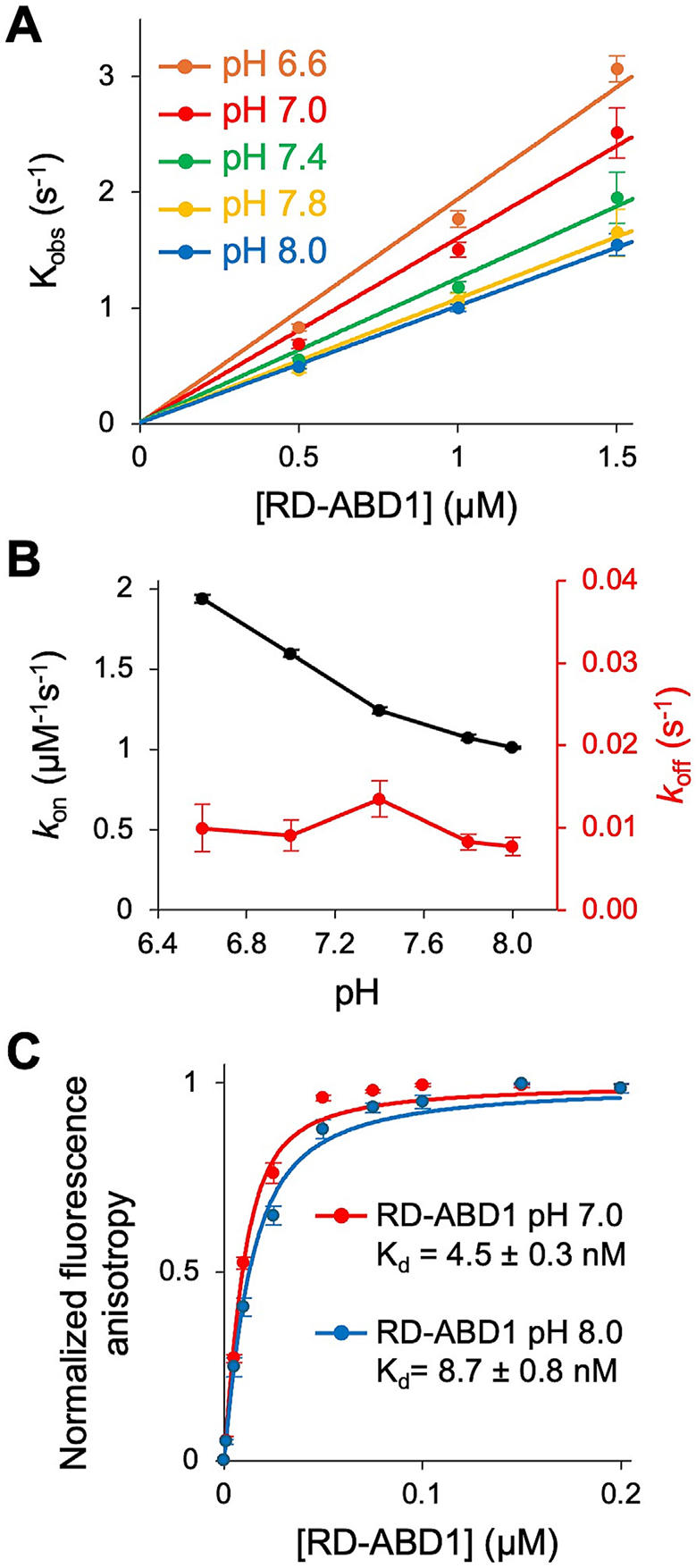
Inhibitory ABD1-ABD2 association is not responsible for the weaker bundling ability of PLS2 at basic pH. (**A**) Slopes from linear fits of $K_{obs}$ values (symbols) determined from kinetic association experiments of FM-ABD2 with RD-ABD1 were used to obtain $k_{on}$ values for various pH conditions (Table S3); error bars represent SD of the mean (*n* ≥ 8). (**B**) $k_{on}$ (black) and $k_{off}$ (red) plotted as a function of pH. $k_{on}$ values derived from the slope of the linear function fit to experimentally measured $k_{obs}$ data shown in (A), error bars represent curve fit error. $k_{off}$ values derived from dissociation experiments of FM-ABD2 from RD-ABD1 with excess unlabeled ABD2, fit to a single exponential. Error bars represent SD of the mean (*n* ≥ 4). (**C**) Equilibrium binding data (symbols) of FM-ABD2 binding to RD-ABD1 at pH 7.0 (red) and 8.0 (blue) fit to a quadratic isotherm (lines). Error bars represent the SD of the mean (*n* = 3). $K_{d}$ values are reported as the mean ± SD from three technical replicates.

The inhibitory association between ABDs is a key regulatory mechanism in plastins [29,30]. To test whether the detected pH sensitivity is related to this mutual inhibition, we measured the effect of pH on the binding kinetics of RD-ABD1 and ABD2 (Figure 3A-C). The dissociation rates of the complex, $k_{off}$, did not change significantly across the explored pH conditions, fluctuating around 0.008 s^−1^ (Figure 3B, Table S3). The association rates slowed moderately at higher pH, reaching ~2-fold difference between pH 6.6 and 8.0 (Figure 3A, B, Table S3). $K_{d}$ derived from kinetic rates reasonably agreed with those measured directly in equilibrium experiments, revealing ~2-fold stronger association at pH 7.0 than pH 8.0 (Figure 3C, Table S3). While the interaction between RD-ABD1 and ABD2 is mildly pH-sensitive, it favors weakening the inhibitory ABD1-ABD2 association [29] at the elevated pH (Table S3). If this were the mechanism primarily responsible for the pH sensitivity of PLS2, it should result in stronger bundling at basic pH, which is not the case. Therefore, the observed pH sensitivity of the ABD1-ABD2 association is unlikely to account for the weaker bundling capacity of PLS2 at basic pH.

### Screening mutagenesis revealed His207 as a potential pH sensor

Changes in protein properties near neutral pH are commonly mediated by changes in the protonation state of His residues [64], whose ${\text{pK}}_{a}$ values fall in this range. Histidines are responsible for pH sensitivity of talin, cofilin, FAK, and AIP1 [8,15,16,18]. To explore the role of specific His residues in the pH dependence of F-actin bundling by PLS2, we used His-to-Lys and His-to-Tyr mutations. The former substitution mimics the charge of protonated His (favored by low pH conditions), while the latter mimics the lack of charges of deprotonated His side chain (favored by higher pH), with the reservation that the selected mutations do not well reproduce histidine geometry and other properties.

We created a library of single His-to-Lys replacements for each of the eight His residues in PLS2 and tested their abilities to bundle actin at pH 7 and 8 (Figure S2). In a preliminary screen with two different ratios of PLS2 to actin, we found that H116K severely inhibited F-actin bundling, regardless of pH (Figure S2A). H116 is located in the linker between RD and ABD1 and neighbors Ser117, whose mutation to Glu, S117E, similarly ablates F-actin bundling by PLS2 (Figure S3). The mechanism by which introducing a charge to His116 or Ser117 inhibits actin bundling is unclear, but the role of this region in actin binding correlates to that of other *t*-CH protein superfamily proteins [61,62]. While PLS2 constructs carrying H207K, H277K, H378K, and H415K mutations also showed aberrations in the pH sensitivity in the preliminary experiments (Figure S2A), upon careful characterization at five different ratios of PLS2 to actin, only H207K satisfied the sought pH-insensitivity [*i.e.*, retained bundling ability at basic pH (Figure S2B)].

### PLS2 H207Y, but not H207K, partially reproduces the mechanisms governing PLS2’s pH-sensitivity

**Figure 4.**
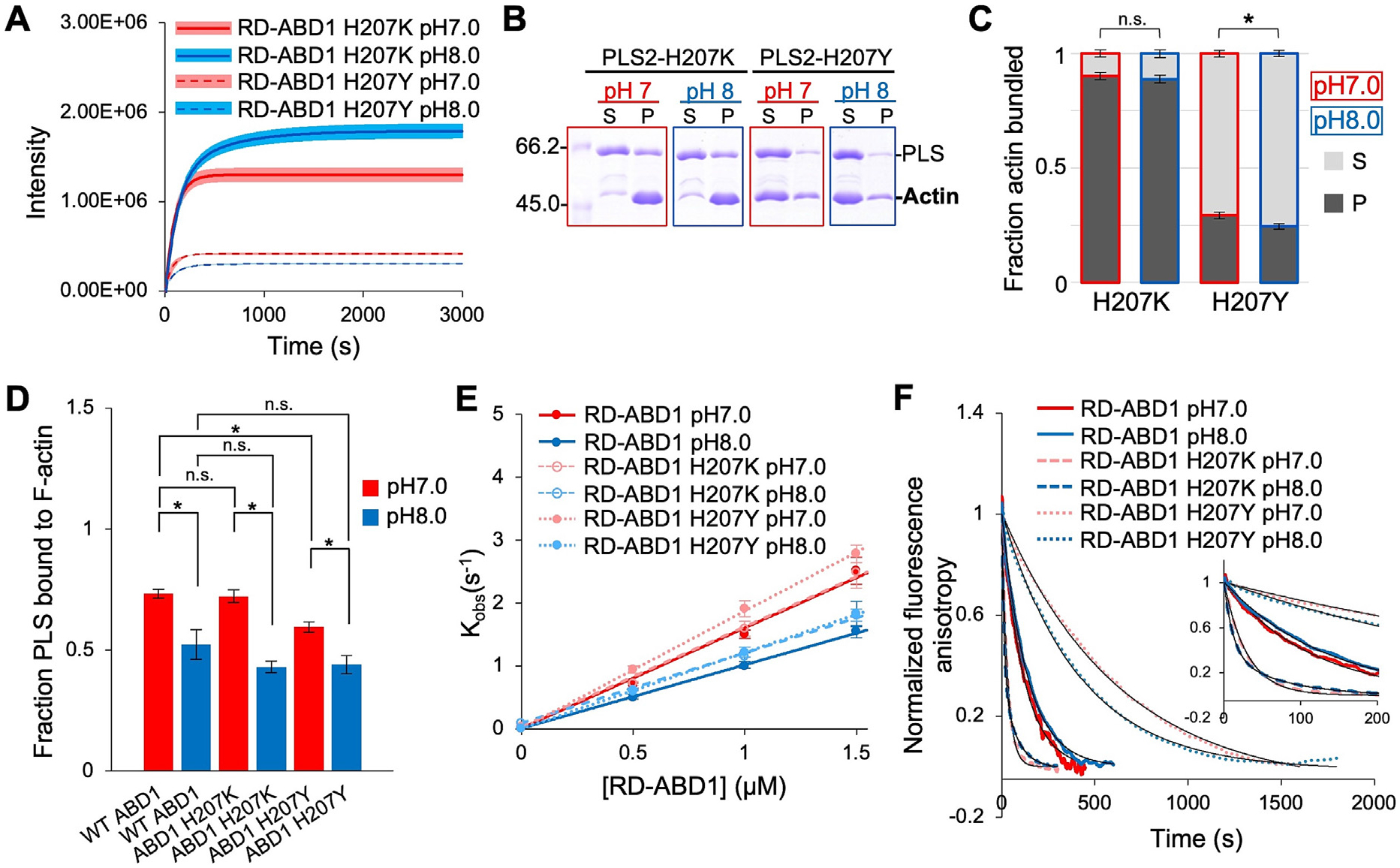
PLS2 H207Y, but not H207K, partially reproduces the mechanisms governing PLS2’s pH-sensitivity. (**A**) Light scattering traces for F-actin bundling by PLS2 mutants at pH 7.0 (red) and pH 8.0 (blue). Lines are averaged extrapolated data, colored areas are SD of the mean (*n* = 3). (**B**) Representative 10% SDS-PAGE gels of supernatant (S) and pellet (P) fractions from low-speed co-sedimentation of actin bundles formed by PLS2 mutants at pH 7.0 and 8.0. (**C**) Quantitation of the low-speed co-sedimentation data. Error bars represent SD of the mean (*n* = 3; *, *P* = 0.003). (**D**) Fraction of ABD1 constructs pelleted during high-speed co-sedimentation with 100 μM F-actin at pH 7.0 (red) and pH 8.0 (blue). Error bars are SD of the mean (*n* = 3). Significance determined by ANOVA analysis is shown in Table S4. (**E**) Slopes from linear fits of $k_{obs}$ values (symbols) determined from kinetic association experiments of FM-ABD2 with the indicated RD-ABD1 constructs were used to obtain $k_{on}$ values for pH 7.0 and 8.0 (Table S5); error bars represent SD of the mean (*n* ≥ 8). (**F**) Dissociation kinetics traces (color curves) of FM-ABD2 from the indicated RD-ABD1 were averaged (*n* ≥ 8) and fit to a single or double exponential (black curves) to yield $k_{off}$ values for each pH condition (Table S5). Inset shows first 200 s of the time-courses for each experiment.

Light scattering and low-speed co-sedimentation experiments showed that PLS2 H207K bundled F-actin effectively and similarly at both pH 7.0 and 8.0, except that the maximum light scattering intensity for H207K at pH 8.0 was even higher than at pH 7.0 (Figure 4A-C). Conversely, H207Y bundled F-actin poorly at pH 7.0 and 8.0 (Figure 4A-C). Notably, H207Y remained sensitive to pH, as judged by more actin in the pellet and a higher light scattering signal at pH 7.0 than 8.0 (Figure 4A-C), indicating that residues other than H207 are involved in the pH sensitivity of PLS2.

Testing individual ABD1 constructs carrying the same mutations revealed that ABD1-H207Y bound actin weaker than WT ABD1 at pH 7.0 (Figure 4D), supporting that H207 contributes to tuning the interaction strength in a pH-dependent manner. Yet, at basic pH, both H207Y and H207K constructs bound to F-actin similarly weaker, *i.e.*, demonstrated pH sensitivity similar to that of WT ABD1 (Figure 4D; Table S4). This observation also suggests that residues other than H207 contribute to this sensitivity. Accordingly, the inability of the H207K mutation to enhance ABD1 binding at basic pH suggests that it potentiates bundling via a different mechanism.

To check whether the enhanced F-actin bundling by H207K may be due to the altered interaction between the actin-binding domains, the $k_{on}$ and $k_{off}$ rates of FM-ABD2 interaction with RD-ABD1-H207K and RD-ABD1-H207Y constructs were measured. While RD contributes little to binding of ABD1 to actin [27], its contribution to binding to ABD2 is measurable [29], justifying its addition to the ABD1 constructs. The mutations had little to no (less than 1.2-fold) effect on the $k_{on}$ values at both pH 7.0 and 8.0 (Figure 4E; Table S5), but notably affected the dissociation rate constants, $k_{off}$ (Figure 4F; Table S5). Specifically, H207Y decreased $k_{off}$ by a factor of 3–4, while H207K accelerated the dissociation by a factor of 4–12 (Figure 4F; Tables S3, S5). The large 4–12-fold gap reflects the double-exponential character of the dissociation rate of RD-ABD1-H207K at pH 8.0 with 62% and 38% amplitudes, suggesting that basic pH enables two distinct binding modes between RD-ABD1-H207K and ABD2 (Figure 4F; Table S5).

Taken together, stronger bundling of F-actin by PLS2-H207K at basic pHs stems mainly from the greater rate of dissociation of ABD2 from RD-ABD1. This allows PLS2 to populate the bundling-competent state with weakly connected ABD1 and ABD2 more frequently, resulting in an actin-bundling protein with lower sensitivity to pH. The weaker bundling capacity of PLS2-H207Y stems from two separate mechanisms. First, this mutation inhibits the dissociation of RD-ABD1 from ABD2, strengthening the complex and preventing PLS2 from adopting the bundling-competent state. Second, H207Y lowers the affinity of ABD1 to actin, partially reproducing the pH sensitivity mechanism of WT PLS2. Combined, these effects cause the inhibition of actin bundling by PLS2-H207Y, even at pH 7.0.

### PLS2 and PLS3 association with cellular F-actin structures is reduced by basic pH_i_

To assess whether PLS2 shows pH-dependent behavior in the cellular context, we performed live and fixed cell imaging of XTC fibroblasts transiently transfected with mEmerald-tagged PLS2 constructs, while manipulating pH_i_ via nigericin clamping [65]. The pH_i_ of the cells was evaluated using ratiometric imaging of the genetically encoded pH sensor mCherry-SEpHluorin in nigericin-containing buffers adjusted to specific pH values [66]. The generated calibration curve was linear in the tested range of pH 6.5–8.0 and confirmed that pH_i_ before clamping (*i.e.*, under normal cell culture conditions in the absence of nigericin) was ~7.4 (Figure S4A, B).

Live-cell imaging revealed that under pre-clamping conditions, both WT and phosphomimic S5D PLS2 showed diffuse cytosolic localization with enrichment at the cell edge, while also weakly localized at FAs and stress fibers (Figures S5A-D and S4C; Videos S6 and S7) in agreement with our previous findings [48]. When pH_i_ was decreased to 6.5 via nigericin clamping, the majority of PLS2 was redistributed to FAs and stress fibers. Conversely, when pH_i_ was increased to 8.0, PLS2’s association with cellular F-actin structures decreased. Importantly, the observed effects of PLS2 redistribution are not due to the global actin cytoskeleton rearrangements, as mCherry-β-actin distribution and morphology were only marginally affected by the changes in pH (Figure S5A-D; Videos S6 and S7).

We also tested whether weakening the pH sensitivity via H207K or H207Y mutations affects the cellular localization of PLS2. H207K and H207Y PLS2 variants reproduced the cellular localization of WT and S5D PLS2 at low and high pH_i_, respectively, while remaining largely insensitive to pH. In agreement with its higher actin-bundling capacity, H207K strongly decorated F-actin cellular structures under each tested pH_i_ condition (Figures S5E, F and S4D; Video S8). H207Y showed mostly diffuse distribution with negligible pH-independent co-localization with actin-rich structures (Figure S5G, H; Video S9). These data exclude a potential pH-sensitive contribution of the mEmerald tag and confirm that the pH-dependent redistribution of WT and phosphomimetic S5D PLS2 in nigericin-clamped cells under acidic and basic pH can indeed be attributed to the pH-sensing properties of PLS2.

**Figure 5.**
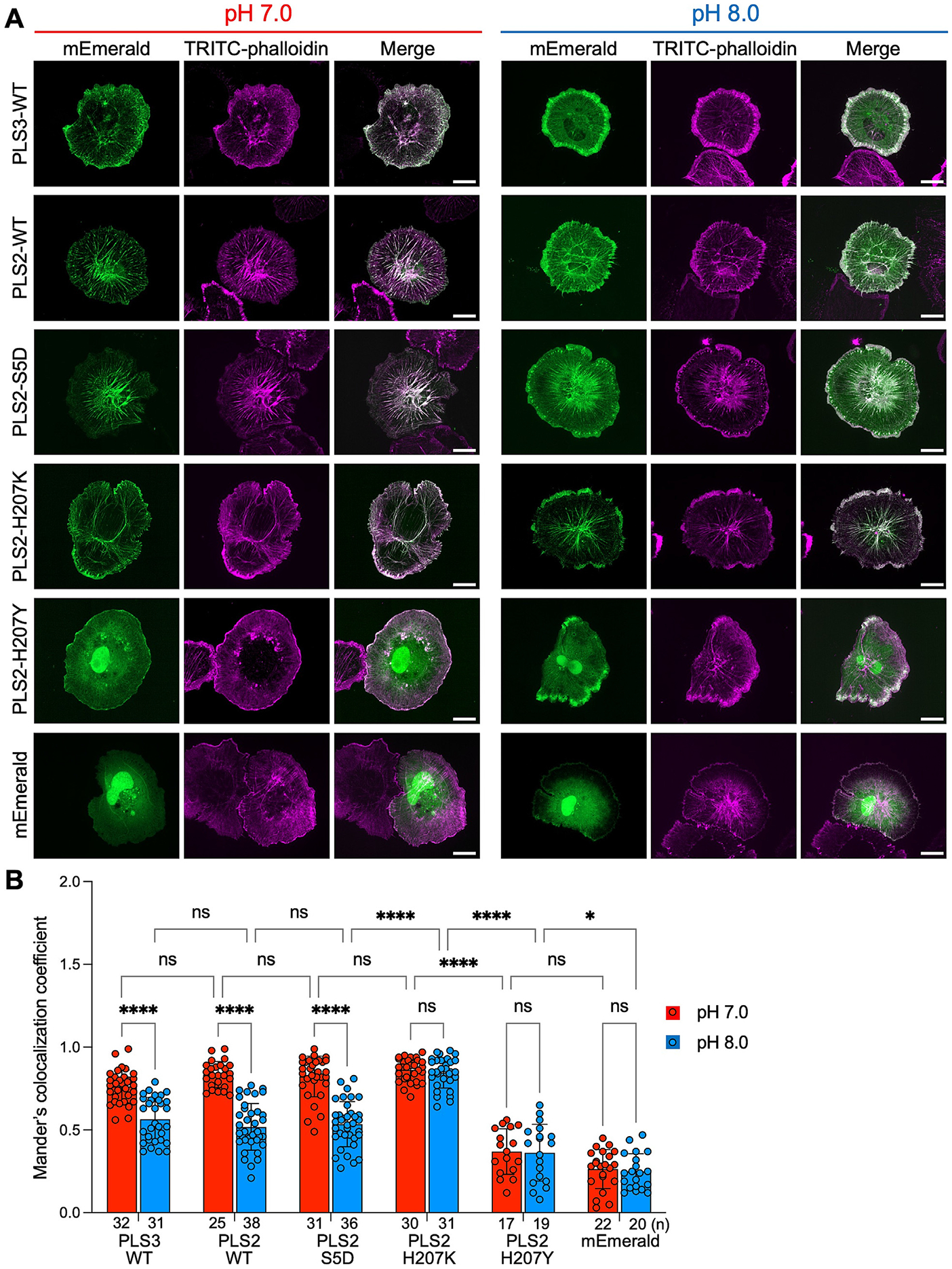
PLS2 and PLS3 show pH-dependent cellular localization. (**A**) Multiple intensity projections of representative confocal images of XTC cells transfected with mEmerald-tagged PLS constructs (green). Before imaging, cells were clamped in nigericin buffers at pH 7.0 or 8.0, fixed, and counterstained with TRITC-phalloidin for F-actin (magenta). Scale bars are 20 μm. (**B**) Colocalization analysis of a fraction of the mEmerald-tagged PLS constructs colocalizing with F-actin (labeled by TRITC-phalloidin) using Mander’s colocalization coefficient (MCC). Data are presented as mean ± SD; dots represent individual data points; number of analyzed cells (*n*) is indicated on the graph. Significance was determined by two-way ANOVA analysis followed by Tukey’s multiple comparisons: ns, non-significant; *, *P* < 0.0333; ****, *P* < 0.0001.

To quantitatively assess the observed pH-dependent intracellular redistribution of plastins, transfected cells were fixed after clamping in nigericin buffers of pH 7.0 or 8.0 and counterstained with tetramethylrhodamine isothiocyanate (TRITC)-phalloidin to label F-actin. Colocalization analysis using Mander’s colocalization coefficient (MCC; Figure 5) corroborated our live-cell results (Figures S4 and S5; Videos S6-S9) and showed that colocalization of mEmerald-tagged WT PLS3 and both WT and S5D PLS2 with F-actin was significantly reduced at alkaline pH, compared to that at neutral pH. Mutating H207 in PLS2 resulted in loss of pH sensitivity, with H207K substitution leading to constitutively high F-actin colocalization level, while H207Y resulting in constitutively low level of colocalization (Figure 5).

It should be noted that nigericin is recognized to perturb Ca^2+^ signaling in various cells, which, given the plastins’ sensitivity to Ca^2+^ [27,48], could offer alternative interpretations of the observed effects. However, in the absence of extracellular Ca^2+^ (*i.e.*, as in our experiments), a rise in the intracellular calcium level ([Ca^2+^]_i_) caused by nigericin is rapid and transient, dropping back to the original level within <2 min post nigericin addition [67,68]. Since imaging in our experiments was initiated after 2 min post nigericin addition and continued for 25 min (Videos S6-S9), the observed effects are unlikely to be related to changes in [Ca^2+^]_i_. Moreover, nigericin-aided pH_i_ alkalinization decreases [Ca^2+^]_i_, while acidification raises it [68-71]. If our results were to be dominated by changes in [Ca^2+^]_i_ rather than pH_i_, we should expect opposite to the observed effects, because high [Ca^2+^]_i_ favors dissociation of plastins from F-actin structures [48].

**Figure 6.**
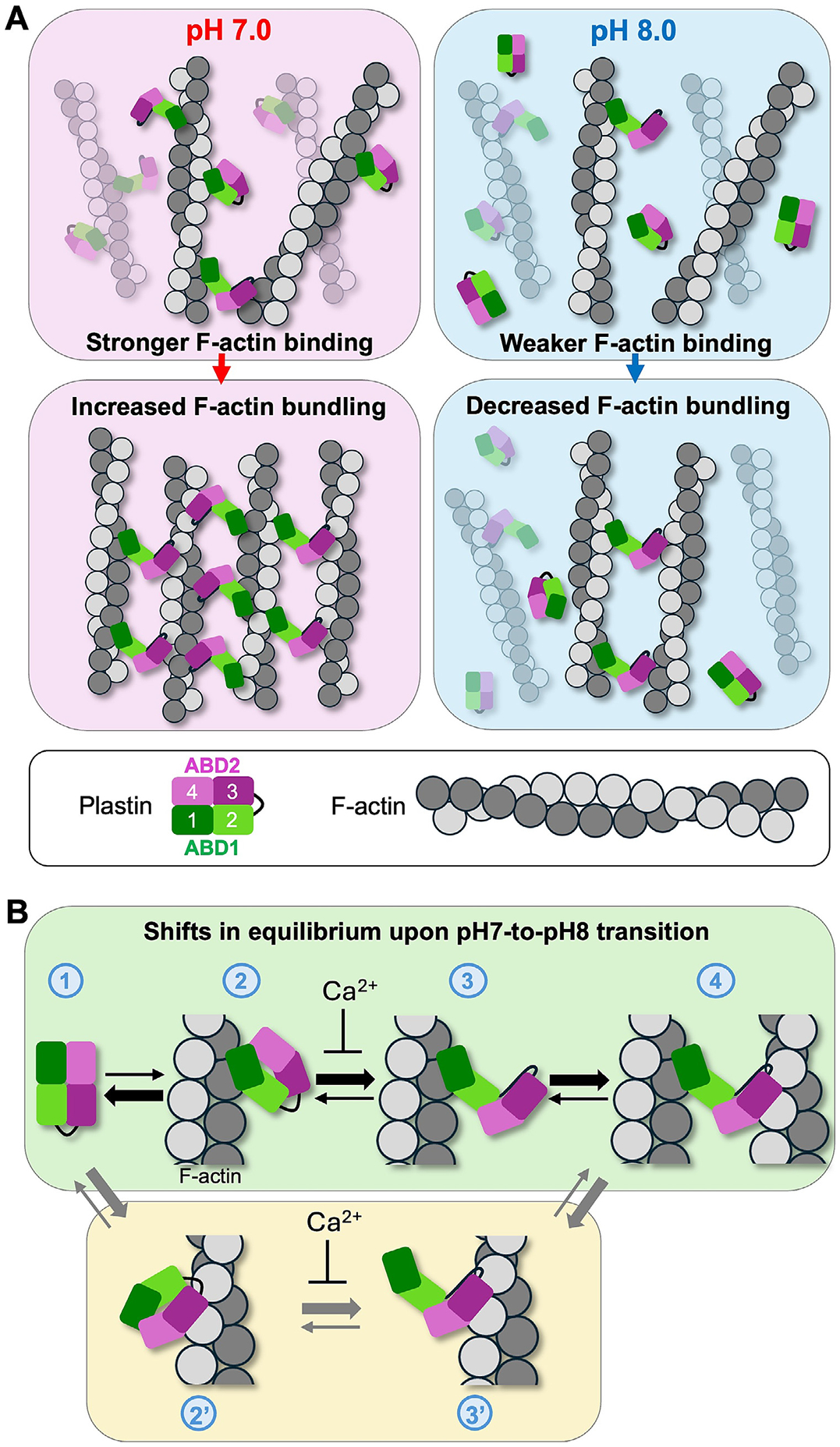
Hypothetical model of pH effects on F-actin bundling by plastins. (**A**) Plastin’s ability to bundle F-actin is regulated by pH: by respectively promoting and inhibiting the binding of ABD1 to actin (top panels), neutral pH enhances F-actin bundling by PLS, while basic pH reduces bundling (bottom panels). (**B**) Shifts (represented by thick arrows) in equilibrium states of plastin upon interaction with F-actin during the transition from pH 7.0 to pH 8.0. Transition from free (state 1) to actin-bound state via ABD1 (state2) is disfavored by basic pH. This binding favors the opening of the ABD core (favored by basic pH), enabling ABD2 to sample the space and bind another actin filament (state 3; favored by basic pH), resulting in bundling (state 4). In the alternative scenario (route 1–2′–3′–4), ABD2 binds first (state 2′; favored by basic pH), leading to a partial release of the ABD1-ABD2 inhibition (state 3′, favored by basic pH), followed by ABD1 binding to actin, forming bundles (state 4; disfavored by basic pH). Notice, however, that this latter scenario implies a stronger binding of PLS to actin at alkaline pH, which is not supported by our experimental data (Figure 1F).

To summarize, under cellular conditions, PLS2 and PLS3 mainly recapitulate their pH-regulated behavior observed *in vitro*: neutral pH_i_ increases the association of PLS2 and 3 with F-actin, while alkalization of pH_i_ decreases it (Figure 6A). Together, our data show that PLS2 and PLS3 are *bona fide* pH-sensing proteins with tunable actin-bundling activity in the physiologically relevant pH range.

## Discussion

In this study, we introduce PLS2 and PLS3 as novel pH-sensitive cytoskeletal proteins with strongly inhibited F-actin bundling activity at basic pH (Figure 6A). We observed a sharp transition towards inhibition of actin bundling by PLS2 at pH > 7.6 (Figure 1B-D). These pH values are substantially higher than the typical cytosolic values of ~7.2–7.4 but similar to the higher local pH values measured at focal adhesions (~7.5–7.7) [6,8] and global pH_i_ measured during the G2/M transition of the cell cycle (~7.6–7.9) [2]. In both processes, the actin cytoskeleton undergoes large-scale rearrangements that likely involve many actin-binding proteins. The increased cytosolic pH coinciding with these rearrangements is consistent with the known roles of talin, cofilin, FAK, cortactin, and AIP1 in favoring actin disassembly and promoting actin dynamics at basic pH [8,15-18,24]. Therefore, the inhibition of actin bundling by PLS2 and PLS3 at basic pH is consistent with plastins participating in changes in actin dynamics during cell migration and cell division.

The current understanding of the mechanism of F-actin bundling by plastins is summarized in Figure 6B. In solution, ABDs of plastins exist in a tight autoinhibited association (Figure 6B, state 1) characterized by a nanomolar affinity even when the domains are mixed *in trans* [29]. Despite that ABD2 affinity to actin is also very high (in a low nanomolar $K_{d}$ range) [29], due to the above ABDs’ autoinhibition, FL plastins interact with actin weakly, and the interaction is primed via only one of the domains, whose identity is debated (states 2 and 2′) [27,29,30,49]. Binding of Ca^2+^ to EF-hands affects the RD interaction with the loop region between ABD1 and ABD2 [48], either preventing the release of the autoinhibition [transition between states 2 (2′) and 3 (3′)] or sterically blocking the association of the secondary ABD with actin. In the absence of Ca^2+^, the interaction between actin and the primary ABD weakens the autoinhibition [29], moderately promoting domain separation, and thus enabling the other ABD to sample the space in search for another actin filament [states 3 (3′)], resulting in bundling (state 4). Our kinetic and equilibrium characterization of PLS2 revealed that basic pH (1) weakens the interaction of ABD2 with RD-ABD1 by a factor of ~2 (Figure 3; Table S3), (2) enhances the interaction of ABD2 with F-actin by ~3-fold (Figure 2E-G; Table 1), and (3) reduces the interaction of ABD1 with F-actin (Figure 2D). Since the first two effects should potentiate bundling (*i.e.*, by favoring the transition through states 2′ and 3′–4, or from 3 to 4), the observed reduction of the PLS2 bundling ability at basic pH appears to be dominated by a weakened affinity of ABD1 to actin. Such domination can be manifested either by hindering the 1–2 state transition, if ABD1 is the primary actin-binding domain (Figure 6B, green panel), or the transition from state 3′ to state 4, if ABD2 is the primary domain (Figure 6B, yellow panel). However, the latter case should imply a stronger binding of PLS2 via ABD2 under basic pH, which is not the case. Indeed, the pH dependence of FL PLS2 binding to actin reproduces that of ABD1, as both bind weaker at basic pH (Figures 1F and 2D), and not that of ABD2, which is stronger at pH 8.0 (Figure 2E-G). Together, these data favor the “ABD1 binds first” hypothesis (Figure 6B, green panel), consistent with our previous observations [27,29]. While ABD1 has orders of magnitude lower affinity for actin than ABD2, its binding in the inhibited state may be favored by a polymorphic nature of this interaction, as judged from the difference revealed from cryo-EM reconstructions of actin filaments decorated by isolated ABD1 [29] and actin bundled by FL PLS3 [30].

In screening for pH sensor residues, we identified His207 as a likely candidate whose mutagenesis reasonably reproduced the pH-sensitive variations in PLS2 activity. Yet, the introduced constitutively protonated (H207K) and deprotonated (H207Y) residues appear to only partially mimic the pH-sensing mechanisms of WT PLS2. Indeed, H207Y mimicked basic pH effects by moderately inhibiting the interaction between actin and ABD1 at neutral pH (state 1–2 (2′) transition on Figure 6B), but it also strengthened the ABD1-ABD2 binding [*i.e.*, inhibited the transition 2 (2′)–3 (3′)], reproducing the effects of acidic pH on this interaction. H207K caused no influence on the pH sensitivity of ABD1-actin interaction but reduced the affinity of ABD1 to ABD2. Thus, the H207K mutation does not affect the transition from state 1–2 (2′) but facilitates the separation of ABDs and the transition from state 2 to 3 (or 2′–3′), thus favoring the bundling (state 4). Of note, the weakening of the inhibitory ABD1-ABD2 association by H207K likely recapitulates that upon the phosphorylation/mutation of S406 [29]. This similarity should not be surprising given that, despite being located in two different ABDs, H207 and S406 are only 4.2 Å apart (Cα–Cα distance) in the AlphaFold2 structure of PLS2 (Figure S6A, B). While not fully recapitulating the pH sensitivity mechanisms of WT PLS2, the net effects of H207K and H207Y mutations mimic the effects of acidic and basic pH_i_, respectively, and, as such, are valuable as tools for studying the role of pH in PLS2 regulation (Figures 5, S4, and S5). It is worth noting that H207 residue is conserved in PLS3, and its respective mutations (H210K and H210Y) will most likely recapitulate the phenotypes observed for H207 mutants of PLS2.

While searching for residues mediating pH-sensing, we found that H116K substitution in PLS2 strongly inhibits F-actin bundling (Figure S2A), just as the S117E mutation of the neighboring residue (Figure S3), suggesting that neither positive nor negative charges are tolerated at this position. Interestingly, the homologous PLS3 residues in a cryo-EM structure of plastin-crosslinked F-actin bundle are disordered and do not obviously contribute to actin binding [30]. In the AlphaFold2-predicted PLS2 structure, these residues form a small anti-parallel β-sheet by bonding with residues A102 and I103 (Figure S6C-E). Whether this region of PLS2 contributes to actin binding directly as the N-terminal residues preceding the *t*-CH domain of β-III-spectrin [62], or affect binding indirectly by changing the thermodynamic stability of PLS2 as it was proposed for utrophin [61], or by affecting the regulation of PLS2 by RD, remains to be established.

Whereas actin itself can be pH sensitive, we focused on plastins rather than on actin as the likely source of the observed pH-dependent changes for the following reasons. For other pH-sensing actin partners, namely, ADF/cofilins, talin, gelsolin, and villin, the sensitivity is encoded in their sequences and involves histidine as at least one of the sensor residues [14,16,72,73]. The advantage of such a design is that the pH sensitivity for various partners can be tuned to a particular sensitivity range or kept unaffected for many other partners. Yet, we cannot exclude the possibility that actin functions as a pH sensor in the pair actin/plastin. Indeed, two actin residues, His87 and His88, are located near the ABD/F-actin binding interface [29,30]. However, their predicted pK_a_ values are between ~5.6 and 6.2 (PROPKA analysis [74]), which makes them unlikely candidates for mediating the observed changes in the pH 7.4–7.8 range.

Endogenous expression of PLS2 is restricted to hematopoietic cells [37], where it contributes to the formation and stabilization of immune synapses [75,76], podosomes [77,78] and sealing rings [79], but also to the migration of various immune cells [55,80]. Immune cell migration is known to be inhibited by an acidic environment, a common inflammation hallmark [81]. One recognized mechanism of this inhibition is a stronger association of integrins with the extracellular matrix at acidic pH [82]. However, the metabolic drop of the extracellular pH is accompanied by intracellular acidification [83], and, therefore, the inhibited migration may also be mediated by pH-sensitive proteins. For PLS2, its stronger bundling capacity in the acidic environment may promote the stability of adhesive structures such as podosomes, which are known to stabilize in response to the acidification of the osteoclast’s cytoplasm [84,85]. While the potential implications of this regulatory mode are numerous, further studies are needed to clarify the role of PLS2’s pH sensitivity in regulating the activity of immune cells.

Intracellular pH is constitutively elevated in cancer [3], where it enhances proliferation, migration, and invasion [86]. A key requirement for mesenchymal cell migration is the ability to regulate FA turnover [87]. This is achieved by the activation of NHE1, leading to a localized rise in pH_i_ that promotes the disassembly of FAs [6,8,16]. Both nascent FAs at the leading edge and mature FAs in the cell interior must be regulated to ensure proper migration. PLS2 is abnormally expressed in many cancers [39-41], where its presence correlates with their boosted invasive and metastatic properties. Similarly, PLS3 is implicated in cell migration [88,89] in both healthy and cancerous tissues [45,90]. Our findings suggest that the pH-sensitivity of PLS2 and PLS3 allows these proteins to respond to increased pH_i_ (*e.g.*, at FAs) by weakening actin bundles and allowing depolymerization factors, such as cofilin and AIP1, to sever and disassemble actin filaments.

Under physiological conditions, many actin partners work together or compete to form actin bundles and meshworks [91]. How their cooperation is orchestrated at the cellular level is poorly understood. Thus, fascin is a ubiquitous actin bundling protein that spatially overlaps with plastins in focal adhesions, cell cortex, lamellipodia and filopodia, as well as in podosomes/invadopodia, and stereocilia of inner ear hair cells [91]. Actin bundles formed by fascin are always parallel [92], which contrasts with the plastin-produced bundles that may contain filaments in both parallel and anti-parallel orientations (this study and [30,56]). Which of the architectures would dominate is likely defined by an intricate interplay of numerous factors. Thus, in the presence of Ca^2+^, the Ca^2+^-independent fascin would dominate. On the other hand, acidic pH would facilitate fascin phosphorylation and inactivation by protein kinase C [93], leading to domination of plastins whose activity is favored under these conditions.

PLS2 and PLS3 belong to the plastin/fimbrin family of actin-bundling proteins, which are part of the larger family of *t*-CH actin-binding proteins, substantially overlapping with the so-called spectrin superfamily [26]. The ABD shared by these proteins has a similar binding footprint on actin [29,30,62,94] and is hypothesized to share the mechanism of “domain-opening,” which regulates their affinity to F-actin [29,30,94-97]. With the herein presented example of plastins having pH-dependent F-actin bundling activity and the previously reported pH sensitivity of *D. discoideum* and *H. pulcherrimus* α-actinins [24,25] it is conceivable that actin binding by other members of the spectrin superfamily may also be sensitive to pH. The hitherto uncharacterized pH sensitivity of these actin-binding proteins may be of considerable importance to disease states such as cancer and neurodegeneration, both of which display dysregulated intracellular pH [3,4].

## Methods

### Protein purification and labeling

Skeletal actin was purified from rabbit skeletal muscle acetone powder (Pel-Freeze Biologicals), as previously described [60]. Actin was stored on ice in G-buffer [2 mM Tris-HCl, pH 8.0, 0.2 mM CaCl_2_, 0.2 mM ATP, 5 mM β-mercaptoethanol (β-ME), 0.005% sodium azide] for no longer than one month with dialysis into fresh G-buffer after two weeks of storage.

QuikChange Lightning Multi-Site-Directed Mutagenesis kit (Agilent Technologies) was used to introduce mutations into plastin constructs cloned in-frame with a tobacco etch virus (TEV) protease recognition sequence downstream of the N-terminal 6xHis-tag in pColdI vector [27]. Truncation constructs were created using NEBuilder HiFi DNA assembly (New England Biolabs). All sequences were verified by Sanger DNA sequencing [Genomics shared resource, The Ohio State University Comprehensive Cancer Center (GSR OSUCCC)]. PLS2-S5D was used as a control in all experiments with PLS2 mutants created on the S5D background (*in vitro* properties of PLS2 are not affected by S5D mutation) [27].

Recombinant proteins were expressed in and purified from BL21-CodonPlus(DE3)pLysS *Escherichia coli* (Agilent Technologies) by immobilized metal affinity chromatography (IMAC) as previously described [98] using HisPur cobalt resin (Thermo Scientific). The 6xHis-tag was removed from all purified proteins by treating them overnight with TEV protease at a 1:20 mol ratio, followed by re-incubation with HisPur cobalt resin to remove the cleaved 6xHis-tag and His-tagged TEV protease. Flow-through fractions containing tagless protein were concentrated and further purified on a Superdex 200 Increase 10/300 GL sizeexclusion column (GE Healthcare) equilibrated with PLS buffer [10 mM 4-(2-hydroxyethyl)-1-piperazi neethanesulfanoic acid (HEPES), pH 7.0, 30 mM KCl, 2 mM MgCl_2_, 0.5 mM ethylene glycol-bis(β-aminoethyl ether)-N,N,N’,N’-tetraacetic acid (EGTA), 2 mM dithiothreitol (DTT)]. Purified tagless PLS constructs were aliquoted, flash-frozen in liquid nitrogen and stored at −80 °C. ABD2 was labeled with fluorescein maleimide (FM; Thermo Fisher Scientific) in PLS buffer devoid of reducing agent at 4 °C as previously described [29].

### Buffer pH determination

The pH values for each buffer condition were carefully controlled via the following procedures. G-buffer was used as the initial solvent, to which all other buffer components (*i.e.*, 10 mM HEPES, 30 mM KCl, 2 mM MgCl_2_, 0.5 mM EGTA) were added from stock solutions to make the PLS buffer with a desired pH measured using a pH Basic meter (Sartorius) with a Fisherbrand Accumet liquid-filled mercury-free pH/ATC electrode (Fisher Scientific). Since the pH of a buffered solution changes in response to ionic strength [99], the initial pH parameters of the 1 M HEPES stock solutions were related to the final pH of the PLS buffer solutions by a standard curve of a linear character (Figure S7). The linear fit yielded Equation (1), which we used to prepare each 1 M HEPES stock solution:

(1) $$pH_{f}=0.768\times pH_{0}+1.60,$$

where $pH_{f}$ is the pH of the final PLS buffer, and $pH_{0}$ is the pH of the 1 M HEPES stock solution. The 1 M HEPES stock solutions were frozen immediately after preparation and used for the preparation of the final buffers, whose actual pH values were experimentally verified using the pH-meter. Refer to the Supplementary Material (Supplementary Methods section) for additional details.

### Light scattering assays

Tagless plastin constructs were cleared by ultracentrifugation at 300,000*g* for 30 min at 4 °C using an Optima MAX-TL ultracentrifuge (Beckman Coulter). The supernatant was collected, and the remaining soluble protein concentration was determined by absorbance at 280 nm using extinction coefficients determined by ProtParam on the Expasy server [100]. G-actin in G-buffer was degassed and placed in UV-transparent glass cuvettes; after five minutes, EGTA and MgCl_2_ were added simultaneously to the final concentrations of 0.5 and 0.1 mM, respectively, mixed, and allowed to incubate for another five minutes to switch from Ca^2+^-bound to Mg^2+^-bound G-actin state. Polymerization was initiated by the addition of the mixture of HEPES, KCl, MgCl_2_, EGTA to the final concentrations specified for the PLS buffer. When bundling was studied, the specified plastin constructs were added at the polymerization initiation time. For experiments in Figures 1B, 2A, S1A, and S1B, the final concentrations of actin and plastin were 10 μM and 5 μM, respectively. Experiments using pre-polymerized actin (corresponding traces in Figure S1H) were conducted in a similar manner, except actin was pre-polymerized as described above but at higher concentration (25 μM) followed by dilution to 5 μM using PLS buffers of pH 7.0 or 8.0, and bundling was initiated by the addition of 2 μM PLS3. To exclude aggregation of plastin as a potential source of the enhanced light scattering, PLS2 and PLS3 (5 μM final concentrations) were analyzed in PLS buffer of pH 7.0 without actin (corresponding traces in Figure S1H). The light scattering intensity change associated with actin polymerization and bundling was measured at 90° to the incident light using a PTI QM-400 fluorometer (Horiba Scientific) with excitation and emission wavelengths set to 350 nm at 25 °C. Measurements were taken every ~10 s, except up to 40 s pauses needed for adding experimental components, which were added to cuvettes in a staggered sequence, with the first and last cuvette initiated at ~10 min and at ~25 min, respectively. Since the resulting asynchronous gaps in the data replicates did not allow direct averaging, the traces fit as single, double, or triple exponentials, and the determined parameters were used to extrapolate missing data and fill the gaps. The extrapolated data was averaged and presented in all light scattering curves. Upon completion of the experiment, samples were pelleted via low-speed co-sedimentation and analyzed by electrophoresis (see below).

### Co-sedimentation assays

Low-speed co-sedimentation assays to monitor F-actin bundling either followed light scattering experiments (as described above) or were conducted as independent experiments. In the latter case, 5 μM actin in G-buffer was incubated with 0.5 mM EGTA and 0.1 mM MgCl_2_ on ice for 5 min, followed by the addition of 1 M HEPES of the desired pH, KCl, and MgCl_2_ up to the final concentrations of 10, 30, and 2 mM, respectively, and allowed to polymerize for at least 30 min at 22 °C. Plastin constructs of various concentrations were mixed with polymerized actin and incubated overnight at 4 °C, followed by further incubation at 22 °C for one hour. The reactions were spun at 20,000 g, 25 °C, for 20 min. Immediately following the centrifugation, supernatants were separated from pellets and resolved by SDS-PAGE.

During high-speed co-sedimentation, actin was incubated with 0.5 mM EGTA and 0.5 mM MgCl_2_ (higher [Mg^2+^] was used due to the high [actin] > 100 μM) on ice for 5 min before the addition of 10 mM HEPES of indicated pH, 30 mM KCl, and 1.5 mM MgCl_2_ to a final concentration of 2 mM. Plastin constructs were mixed with polymerized actin and incubated overnight at 4 °C, followed by additional incubation at 22 °C for at least one hour. The final concentration of plastin in Figures 1E, F, and S1I was 5 μM, while the final concentration of plastin in Figures 2D and 4D was 2 μM. The samples were centrifuged at 300,000*g*, 25 °C, for 30 min in an Optima MAX-TL ultracentrifuge (Beckman Coulter). Supernatants were separated from pellets immediately after the spin, pellets were soaked with equivalent volumes of 1x reducing sample buffer at least for 2 h, collected via vigorous pipetting, and resolved by SDS-PAGE. For both high- and low-speed co-sedimentation, gels were stained with Coomassie brilliant blue R-250, and gel band intensities were quantified by densitometry using ImageJ v.2.3 software [101,102]. The number of repetitions is indicated in the figure legends. Uncropped SDS-PAGE gels are shown in the Appendix A1.

### Stopped-flow kinetics

Time courses of FM-ABD2 binding to or dissociating from F-actin or the indicated RD-ABD1 construct were recorded, and association and dissociation rates were determined by the change in fluorescence anisotropy signal detected by an SX-20 LED stopped-flow spectrometer (Applied Photophysics) at 25 °C. The dead time of the instrument is 1 ms. Samples were excited by a 470 nm LED element (Applied Photophysics), and changes in fluorescence anisotropy were measured using two identical 515 nm long-pass colored glass filters (Newport Corporation) in parallel and perpendicular channels. To determine the association rates of FM-ABD2 (50 or 100 nM) with F-actin or the specified RD-ABD1 construct, these proteins were loaded to the instrument syringes in parallel. The unlabeled proteins were present in a range of concentrations at least 10 times higher than that of FM-ABD2 to ensure pseudo-first-order kinetic conditions. Time courses from the association experiments were individually fit to a single exponential equation using Pro-Data SX and Pro-Data Viewer (Applied Photophysics). All $k_{obs}$ values are the results of at least 8 association time course replicates. Error bars represent the standard deviation (SD) of the mean.

$k_{off}$ values were directly measured by recording the dissociation of FM-ABD2 from either F-actin or the specified RD-ABD1 construct upon competition with excess of unlabeled ABD2. For dissociation time courses of FM-ABD2 and F-actin, either one of two conditions were used: 500 nM F-actin stabilized with 2 μM phalloidin bound to 50 nM FM-ABD2, or 100 nM F-actin stabilized with 1 μM phalloidin and bound to 25 nM FM-ABD2. For each pH and protein concentration condition, 7.5 and 10 μM unlabeled ABD2 were used to compete with pre-bound FM-ABD2. Both concentrations of unlabeled ABD2 yielded similar rates, confirming that the reassociation of FM-ABD2 was not an issue. These concentrations yielded at least 4 dissociation time courses for each pH condition, single exponential fits of which provided the sought $k_{off}$ values.

For dissociation time courses with FM-ABD2 and the specified RD-ABD1 construct, 25 or 50 nM FM-ABD2 was bound to 50 or 100 nM RD-ABD1 and competed with 7.5 to 20 μM unlabeled ABD2. These concentrations yielded at least four dissociation time courses for each concentration of ABD2 at each pH. These time courses were individually fit to a single or double exponential, which yielded rate constants that were averaged and taken as the $k_{off}$. Both the lower and higher concentrations of ABD2 yielded similar results. All $k_{off}$ values for FM-ABD2 with F-actin and FM-ABD2 with RD-ABD1 are reported with standard deviation (*n* ≥ 4).

To determine $k_{on}$, $k_{obs}$ values measured for association time courses were plotted with the experimentally measured $k_{off}$ values and fit to a linear line in GraphPad Prism version 10.0.0 for Windows (GraphPad Software, Boston, Massachusetts USA), which was forced to cross the y-axis at the experimentally measured $k_{off}$. The slope of this line yielded the $k_{on}$ and is reported with curve fit error. Representative curves shown in Figures 2F and 4F are averaged, smoothed curves from at least 4 individual time courses, which were fit to a single or double exponential, normalized, and plotted with their best-fit function.

### Fluorescence anisotropy equilibrium binding assays

The change in fluorescence anisotropy of FM-ABD2 at pH 7 and 8 upon binding to either F-actin or RD-ABD1 was measured using an Infinite M1000 Pro plate reader (Tecan US, Inc.) with excitation and emission wavelengths of 470 and 519 nm, respectively. Fluorescence anisotropy measurements of F-actin binding were performed after equilibration of 5 nM FM-ABD2 with the indicated concentrations of F-actin stabilized with 1.0 μM phalloidin. RD-ABD1 binding measurements were performed using the indicated concentrations of RD-ABD1, equilibrated with 10 nM FM-ABD2. All equilibrium assays were measured after 60 min of incubation at 22 °C, followed by an additional observation after 24 h, to confirm that the equilibrium had been reached. All reactions were carried out in PLS buffer of the indicated pH. The data from three technical replicates were normalized and fit to a quadratic isotherm equation:

(2) $$\frac{S_{i}-S_{min}}{S_{max}-S_{min}}=\frac{P+x+K_{d}\sqrt{{\left(P+x+K_{d}\right)}^{2}-4Px}}{2P},$$

where $S_{i}$ is the signal for each data point, $S_{min}$ is the minimum value for the replicate, and $S_{max}$ is the maximum value from that replicate, P is the concentration of labeled FM-ABD2, and x is the concentration of either F-actin or RDABD1 construct. $K_{d}$ values obtained from equilibrium measurements are the average of three technical replicates, reported with their standard deviations.

### In vitro TIRF microscopy

TIRF microscopy of PLS3-mediated actin bundling was conducted as described [103] with modifications. Due to the observed higher propensity of filaments formed from AlexaFluor 488-labeled actin to adhere to the substrate at pH 7.0 compared to pH 8.0 (which substantially skewed the filament overlap probability), unlabeled actin was polymerized in the presence of TRITC-phalloidin (Millipore Sigma), which resolved this issue. Briefly, G-actin (1.5 μM) was mixed with 50 nM PLS3 and 0.5 μM TRITC-phalloidin in TIRF buffer adjusted to pH 7.0 or 8.0 [final composition: 10 mM imidazole, 0.2 mM EGTA, 1 mM MgCl_2_, 50 mM KCl, 0.25 mM ATP, 10 mM ascorbic acid, 2.5 mM protocatechuic acid (MilliporeSigma, Burlington, MA), 0.1% bovine serum albumin (VWR International, Radnor, PA), 0.6% methylcellulose-400cP (MilliporeSigma, Burlington, MA), and 0.1 μM protocatechuate 3,4-dioxygenase]. The mixture was applied to methoxy-poly (ethylene glycol)-silane 5 K (mPEG-silane; Millipore Sigma) coated TIRF chamber. Time-lapse images (started ~2 min post-mixing) were recorded using Nikon Eclipse Ti-E microscope equipped with a TIRF illumination module (with a 15-mW laser), a Nikon CFI Plan Apochromat γ 100× oil objective (NA: 1.45), a perfect focus system (Nikon Instruments, Melville, NY), and an iXon Ultra 897 EMCCD camera (Andor Technology, Belfast, UK). Comprehensive evaluation of bundling was hampered by high crowding conditions required for actin bundling by plastins in a TIRF chamber. Therefore, we selected bundles for analysis based on the following criteria: (1) a well separated bundle; (2) a thin bundle consisting of 2–4 single filaments; (3) bundle length does not increase during the analyzed period (*e.g.*, anti-parallel bundles or fragments of parallel bundles with the filament barbed ends well separated from each other). The barbed ends were determined by their fast polymerization rate. Note, in an actin filament labeled with TRITC-phalloidin, the fast-growing barbed ends appear less bright than the rest of the filament due to the relatively slow incorporation of phalloidin into the newly formed part of the filament. This contrasts with the conventional AlexaFluor 488-labeled filaments, where barbed ends appear brighter due to the fluorophore bleaching in the rest of the filament. Actin bundles were distinguished from single filaments by brighter fluorescence. The lengths of bundles (or their fragments, in case of bundle unzipping) were measured over time in Fiji/ImageJ2 [101]. The difference between maximum and minimum bundle length (percent unbundled) over the fixed evaluation period (2 min) was expressed as a percent of the maximum bundle length for each analyzed bundle.

### Transmission electron microscopy

G-actin was pre-polymerized at 25 μM concentration followed by dilution to 5 μM using PLS buffers of pH 7.0 or 8.0. Bundling was initiated by the addition of 2 μM PLS2. Samples were incubated for 30 min at room temperature, diluted 5 folds using PLS buffers of pH 7.0 or 8.0, and applied to formvar/carbon-coated grids (Ted Pella, Redding, CA) for 60 s followed by negative staining with 1% (w/v) uranyl acetate for 60 s. The grids were examined using a FEI Tecnai G2 Spirit transmission electron microscope (Campus Microscopy & Imaging Facility (CMIF) at OSU) at an accelerating voltage of 80 kV and a nominal magnification of 34,000×.

### Cell culture and transfections

Xenopus laevis XTC fibroblast cells [104] [obtained from Dr. Watanabe (Kyoto University) and not further authenticated] were cultured in 70% Leibovitz’s L-15 medium (Thermo Fisher Scientific) supplemented with 10% fetal bovine serum, L-glutamine, and penicillin–streptomycin at 23 °C and ambient CO_2_. The cells were mycoplasmanegative as determined by PCR [105]. Transient transfections were performed using Lipofectamine 3000 (Thermo Fisher Scientific) and human PLS2 constructs N-terminally fused to mEmerald [29]. To introduce the desired mutations, mutagenesis was performed using QuikChange Site-Directed Mutagenesis kit (Agilent Technologies). mCherry-β-actin (Addgene #54967, RRID:Addgene_54967) was a gift from Michael Davidson [106]. mCherry-SEpHluorin (Addgene plasmid #32001; RRID: Addgene_32001) was a gift from Sergio Grinstein [107]. Transfected cells were plated on polylysine-coated coverslips (Neuvitro Corporation, Vancouver, WA) in Attofluor chambers (Thermo Fisher Scientific, Waltham, MA) in serum-free L-15 medium and imaged 30 min post-plating.

### pH_i_ clamping and calibration

For pH_i_ calibration curve (Figure S4A, B), cells transiently transfected with mCherry-SEpHluorin were sequentially incubated and imaged in nigericin buffers (10 μM nigericin, 25 mM HEPES, 105 mM KCl, 1 mM MgCl_2_) adjusted to a desired pH using KOH to satisfy the Na^+^-free buffer requirement for equilibration of intracellular and extracellular pH via nigericin clamping [65,66]. Micrographs were obtained using an Eclipse Ti-E inverted microscope (Nikon Instruments Inc., Melville, NY) equipped with a perfect focus system, Nikon CFI Plan Apochromat λ 100x oil objective (NA 1.45), and an iXon Ultra 897 EMCCD camera (Andor Technology, Belfast, UK) using NIS Elements-AR v.4.3 software (Nikon Instruments Inc., Melville, NY). Cells were fragmented using Threshold in Fiji/ImageJ2 [101]. The ratios of the background-corrected fluorescence intensities of SEpHluorin (pH sensor) to mCherry (pH-insensitive reference) were plotted against the corresponding pH value. Data are presented as means ± SD; individual numbers of analyzed cells (n) for each pH condition are given in Figure S4B legend.

Time-lapse imaging (Figure S5; Videos S6-S9) was performed on cells transiently co-transfected with mCherry-β-actin and various mEmerald-PLS2 constructs at intervals of 1 min for 10 min under the pre-clamping conditions. Then, while on the microscope stage, cell medium was replaced with nigericin buffer of a desired pH, and imaging was continued for additional 25 min before changing to a different pH buffer with an additional 25-min imaging. Kymographs and line plot profiles were obtained using KimographBuilder and Plot Profile in Fiji/ImageJ2. In additional experiments, cells were cycled through several changes of nigericin buffers with different pH, and random transfected cells were imaged at each pH_i_ condition tested (Figure S4C, D).

### Colocalization analysis

PLS fraction overlapping with F-actin was analyzed in cells transiently transfected with mEmerald-tagged PLS constructs using Mander’s colocalization coefficients (MCC). 16–24 h post-transfection, the cells were trypsinized, plated on polylysine-coated coverslips, and allowed to spread for 45 min in serum-free L-15 medium, followed by pH_i_ clamping using nigericin buffers of pH 7.0 and pH 8.0 for 15 min. Following clamping, cells were fixed in 2% paraformaldehyde (prepared in buffers of the corresponding pH 7.0 and 8.0; 25 mM HEPES, 105 mM KCl, 1 mM MgCl_2_) and counterstained with TRITC-phalloidin. Confocal z-stack images were obtained using an Eclipse Ti2 microscope (Nikon Instruments Inc., Melville, NY) equipped with Crest X-light V3 spinning disc (CrestOptics, Rome, Italy), ORCA-fusion digital CMOS camera (Hamamatsu Photonics, Shizuoka, Japan), and Nikon CFI Plan Apochromat Lambda D 100x oil objective using NIS Elements AR 6.02.03 software (Nikon Instruments Inc., Melville, NY). Maximum intensity projections (MIP) of the confocal z-stacks were generated using Fiji/ImageJ2. MCCs for background-corrected MIP images were determined using BIOP JACoP Fiji/ImageJ plugin.

### AlphaFold2-generated images

AlphaFold2 [108,109] was used to generate the structure of PLS2 (Figure S6).

### Statistical analysis

When comparing two groups, *p* values were calculated using Student’s *t*-test in Microsoft Excel for Microsoft 365 MSO version 2307 (Microsoft Corporation) (Figures 1E, 2C,D, and 4C) and Prism v. 10.4.2 for MacOS (GraphPad Software, LLC) (Figure 1G, H). All other multiple comparisons were done using analysis of variance (ANOVA) with a Tukey’s post-hoc test in Origin 2023 v.10.0 (OriginLab Corporation), Prism v. 10.0.0 for Windows, or Prism v. 10.4.2 for MacOS. Individual *p* values for the experimental data from Figure 1D are presented in Tables S1 and S2. Individual *p* values for the experimental data from Figure 4D are presented in Table S4. Results were considered significant if the associated *p* value was less than 0.05. The definition of error bars and number of repetitions are described in figure legends. All graphical data is provided in the Appendix A2.

## Supplementary Material

Supplemental Material

Appendix-A1

Video S3

Video S1

Video S2

Video S4

Video S5

Video S6

Video S7

Video S9

Video S8

Appendix-A2_Source data

Supplementary material to this article can be found online at https://doi.org/10.1016/j.jmb.2025.169306.

## Figures and Tables

**Table 1:** Kinetic and equilibrium parameters for association and dissociation of FM-ABD2 to and from F-actin at varying buffer pH. $k_{on}(n\geq 8)$ and $k_{off}(n\geq 4)$ and equilibrium $K_{d}(n=3)$ values are reported as mean ± SD. $K_{d}$ derived from kinetics was calculated using the $k_{off}$ and $k_{on}$ for each specified pH.

pH	$k_{on}(\mu M^{-1}\;s^{-1})$	$k_{off}(s^{-1})$	$K_{d}$ derived from kinetics (nM)	Equilibrium $K_{d}$ (nM)
6.6	0.479 ± 0.006	0.0073 ± 0.0003	15	–
6.8	0.502 ± 0.005	0.0066 ± 0.0002	13	–
7.0	0.600 ± 0.030	0.0057 ± 0.0002	9.5	5 ± 2
7.4	0.601 ± 0.007	0.0073 ± 0.0007	12	–
7.8	1.280 ± 0.020	–	–	–
8.0	1.450 ± 0.040	–	–	1.7 ± 0.3

## Data Availability

The authors declare that the data supporting the findings of this study are available within the article and its Supplementary Material and Appendices files.

## Abbreviations:

ABD: actin-binding domain
AIP1: actin-interacting protein 1
CBM: calmodulin-binding motif
CH: domain, calponin-homology domain
F-actin: filamentous actin
FA: focal adhesion
FAK: focal adhesion kinase
FL: full-length
FM: fluorescein-maleimide
MCC: Mander’s colocalization coefficient
MIP: Maximum intensity projections
NHE1: Na^+^/H^+^ exchanger 1
pH_i_: intracellular pH
PLS: plastin
RD: regulatory domain
*t*-CH domain: tandem calponin-homology domain
TEM: transmission electron microscopy
TIRF microscopy: total internal reflection fluorescence microscopy
TRITC: tetramethylrhodamine isothiocyanate
WT: wild type
