# Supplementary material for: Human Plastins are Novel Cytoskeletal pH Sensors with a Reduced F-actin Bundling Capacity at Basic pH
Source: J Mol Biol. Author manuscript; Available in PMC 2025 Jul 30. (PMC12309418; doi:10.1016/j.jmb.2025.169306)

Supplementary Material for

Human plastins are novel cytoskeletal pH sensors with a reduced F-actin bundling capacity at basic pH

Lucas A. Runyan, Elena Kudryashova, Richa Agrawal, Mubarik Mohamed, Dmitri S. Kudryashov

This file includes:

Supplementary Methods

Supplementary Figures (S1-S7)

Supplementary Tables (S1-S5)

Supplementary Video Captions (S1-S9)

Additional files:

Supplementary Videos (S1-S9)

Appendix A1 (uncropped gels)

Appendix A2 (source data for graphs)

Supplementary Methods

Calculation of ionic strength equivalent for titration of HEPES buffer to pH 8.0

To calculate the ionic strength contributed by the addition of KOH (and the resulting deprotonated HEPES buffer) required to adjust the pH of HEPES buffer from pH 7.0 to pH 8.0, we followed the method described by Ellis & Morrsion [1]. At pH 7.0, 10 mM HEPES (pKa* = 7.39 [1]) buffer solution will be composed of 7.1 mM protonated HEPES (HA, uncharged) and 2.9 mM deprotonated HEPES (A^{-}, negatively charged). Since the concentration of H^{+} and OH^{-} are negligible [1], the total charge of the buffered solution is equal to the amount of strong acid or base required to arrive at the specified pH. The total charge of a pH 7.0 10 mM HEPES is −2.9 mM, which yields an ionic strength of 2.9 mM. Using this same method, the total charge and ionic strength of pH 8.0 10 mM HEPES is −8.8 mM and 8.8 mM, respectively. The difference in ionic strength between these two buffers is 5.9 mM. Therefore, we added 5.9 mM KCl (total of 36 mM KCl) to pH 7.0 PLS buffer to bring it to identical ionic strength with pH 8.0 PLS buffer. Conversely, in a separate experiment, we decreased the concentration of KCl in pH 8.0 PLS buffer to 24.1 mM, to cause the ionic strength of the pH 8.0 buffer to become equal to that of the pH 7.0 buffer.

References:

[1] K.J. Ellis, J.F. Morrison, Buffers of constant ionic strength for studying pH-dependent processes., Methods Enzymol. 87 (1982) 405–26. https://doi.org/10.1016/s0076-6879(82)87025-0.

Supplementary Figures

Figure S1

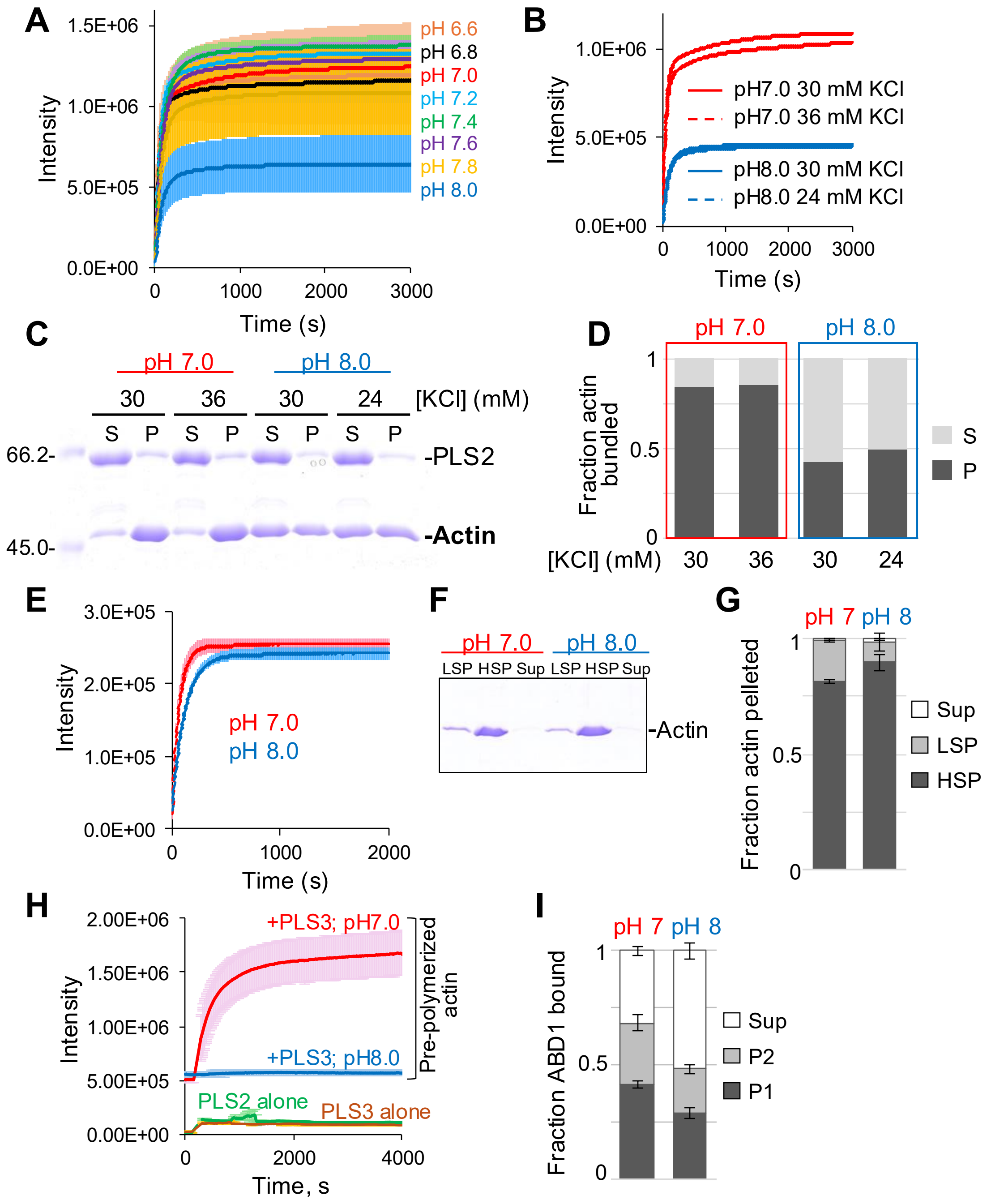

Figure S1. Characterization of pH-dependence of plastins and F-actin. (**A**) Light scattering traces of F-actin bundling by PLS2 at various pH values (some are the same as shown on Fig. 1B). Solid colored lines represent averaged extrapolated data; colored areas represent SD of the mean (n ≥ 3). (**B**) Light scattering traces of F-actin bundling by PLS2 at pH 7.0 and 8.0 under different salt conditions (to account for an additional ionic strength equivalent (6 mM KCl) during buffer pH adjustments (see Supplementary Methods). (**C**) Representative 10% SDS-PAGE gel of supernatant (S) and pellet (P) fractions from low-speed co-sedimentation of actin bundles formed by PLS2 in different pH and salt conditions. (**D**) Fractions of actin in the pellet (P) and supernatant (S) after low-speed co-sedimentation in the presence of PLS2 in different pH and salt conditions quantified from (C). (**E**) Light scattering traces of polymerization of F-actin alone at pH 7.0 and pH 8.0: a full-scale graph of the "Actin alone" traces, which are shown in Fig. 1B. Solid lines represent averaged extrapolated data, colored areas represent SD of the mean (n = 2). (**F**) Representative 10% SDS-PAGE gel of actin fractions from the light scattering experiments (shown in E): LSP, pelleted at low speed; HSP, pelleted at high speed; Sup, supernatant after the high-speed centrifugation. (**G**) Quantitation of the gel (F). Error bars represent SD of the mean (n = 2). (**H**) Light scattering traces of PLS 2 and PLS3 without actin and using pre-polymerized F-actin with PLS3 at pH 7.0 and 8.0. Colored lines represent averaged extrapolated data; colored areas represent SD of the mean (n=2). (**I**) High-speed co-sedimentation experiments of ABD1/F-actin binding: P1 represents fraction of ABD1 present after initial pelleting with 50 μM F-actin; the resulting supernatant was supplemented with another 50 μM F-actin, and pelleted again, yielding another pellet (P2) and a final supernatant (Sup). Error bars represent SD of the mean (n = 3).

Figure S2

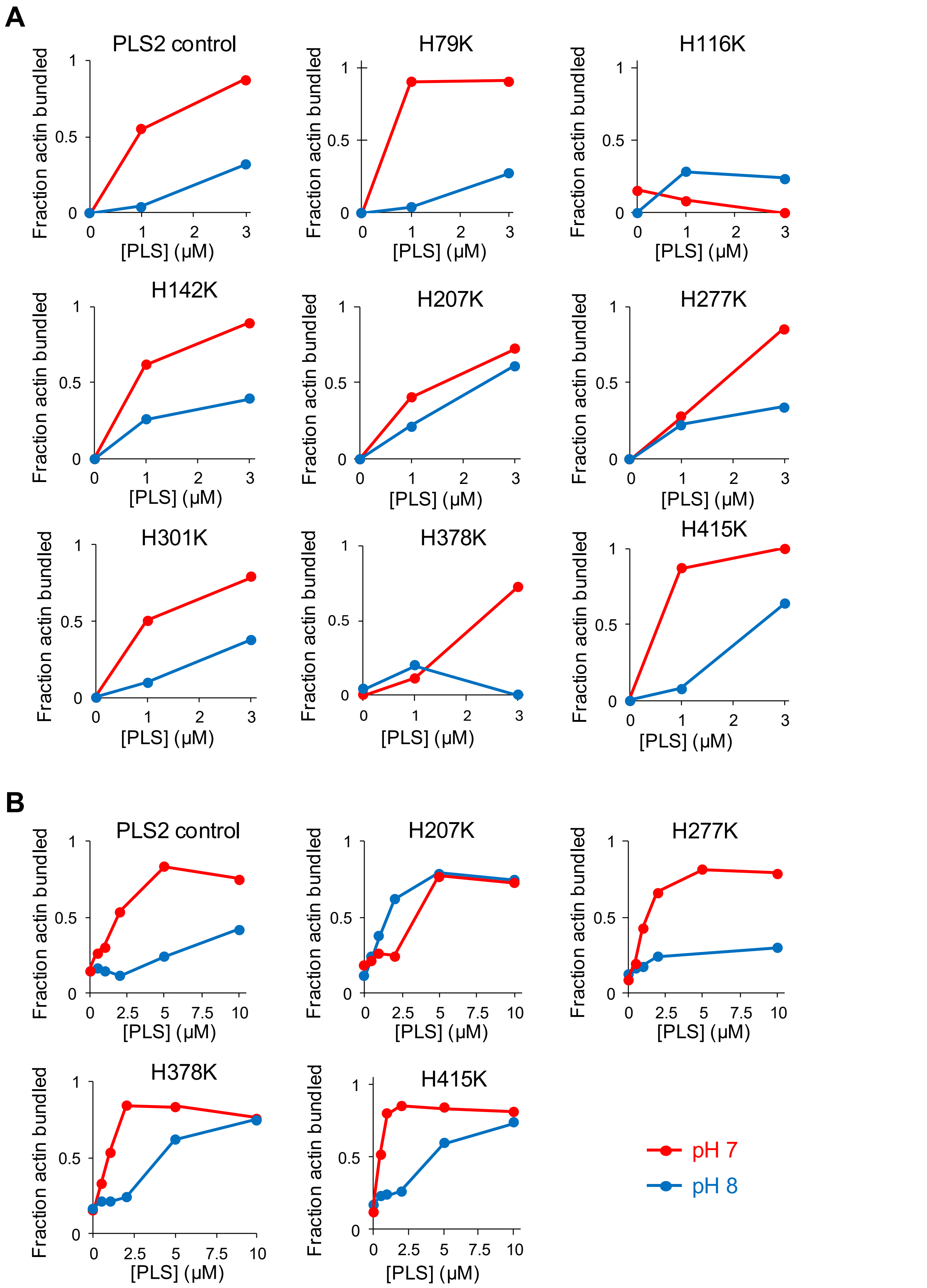

Figure S2. Screening for pH-dependence of F-actin bundling by PLS2 variants with His-to-Lys substitutions. (**A**) Low-speed co-sedimentation of F-actin with indicated PLS2 variants at pH 7.0 (red) and pH 8.0 (blue). (**B**) Additional low-speed actin co-sedimentation experiments of selected PLS2 constructs with additional titration points.

Figure S3

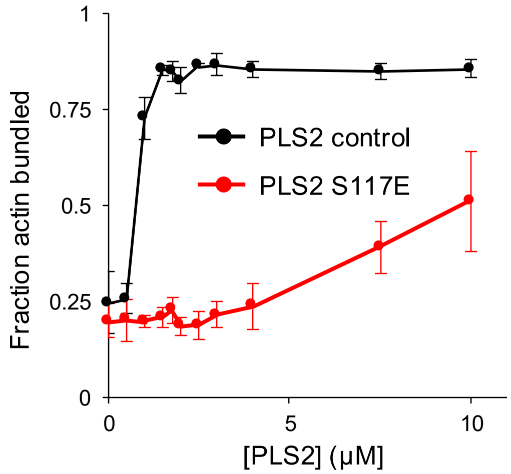

Figure S3. Effect of S117E mutation on F-actin bundling by PLS2 using low-speed actin co-sedimentation. Data are presented as mean ± SD; n = 3 (except for PLS2 control at 2µM, S117E at 0.5, 1.75, and 7.5 µM, where n = 2).

Figure S4

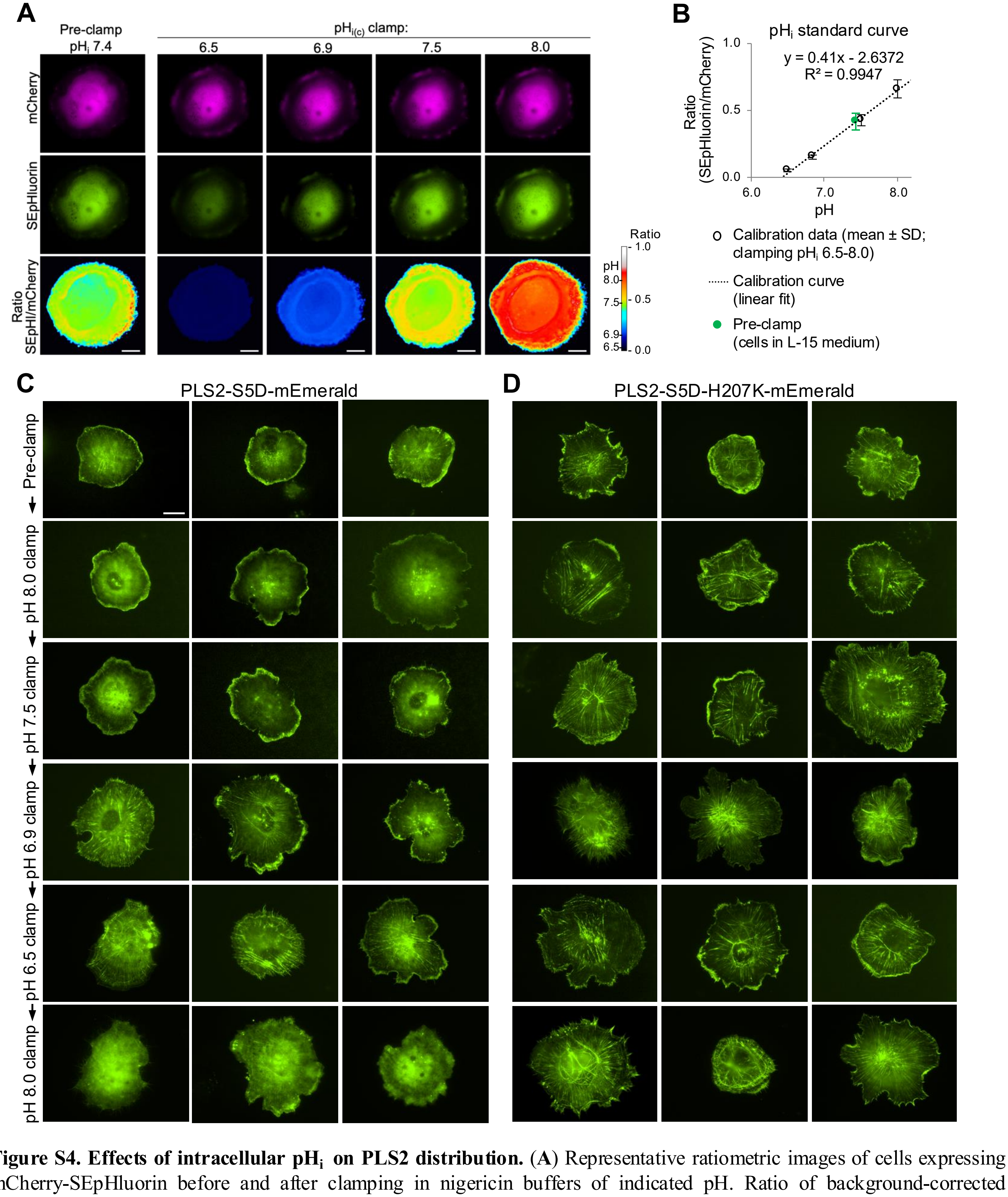

Figure S4. Effects of intracellular pH_i on PLS2 distribution. (**A**) Representative ratiometric images of cells expressing mCherry-SEpHluorin before and after clamping in nigericin buffers of indicated pH. Ratio of background-corrected fluorescence signals in green versus red channels is presented using royal LUT after applying median filter (radius 15 px). Scale bars are 20 µm. (**B**) pH_i standard curve derived from the ratiometric imaging. Data are presented as mean ± SD; number of analyzed cells (n): pre-clamping, n=24; pH_i 6.5, n=29; pH_i 6.9, n=28; pH_i 7.5, n=29; pH_i 8.0, n=27. (**C,D**) Representative images of cells expressing mEmerald-tagged PLS2 constructs (control S5D or S5D-H207K) under different pH_i conditions. Cells were clamped at different pH sequentially (as shown by arrows on the left side). Three individual random cells are shown for each pH_i condition for each construct. Scale bar is 20 µm (identical for all images in C and D).

Figure S5

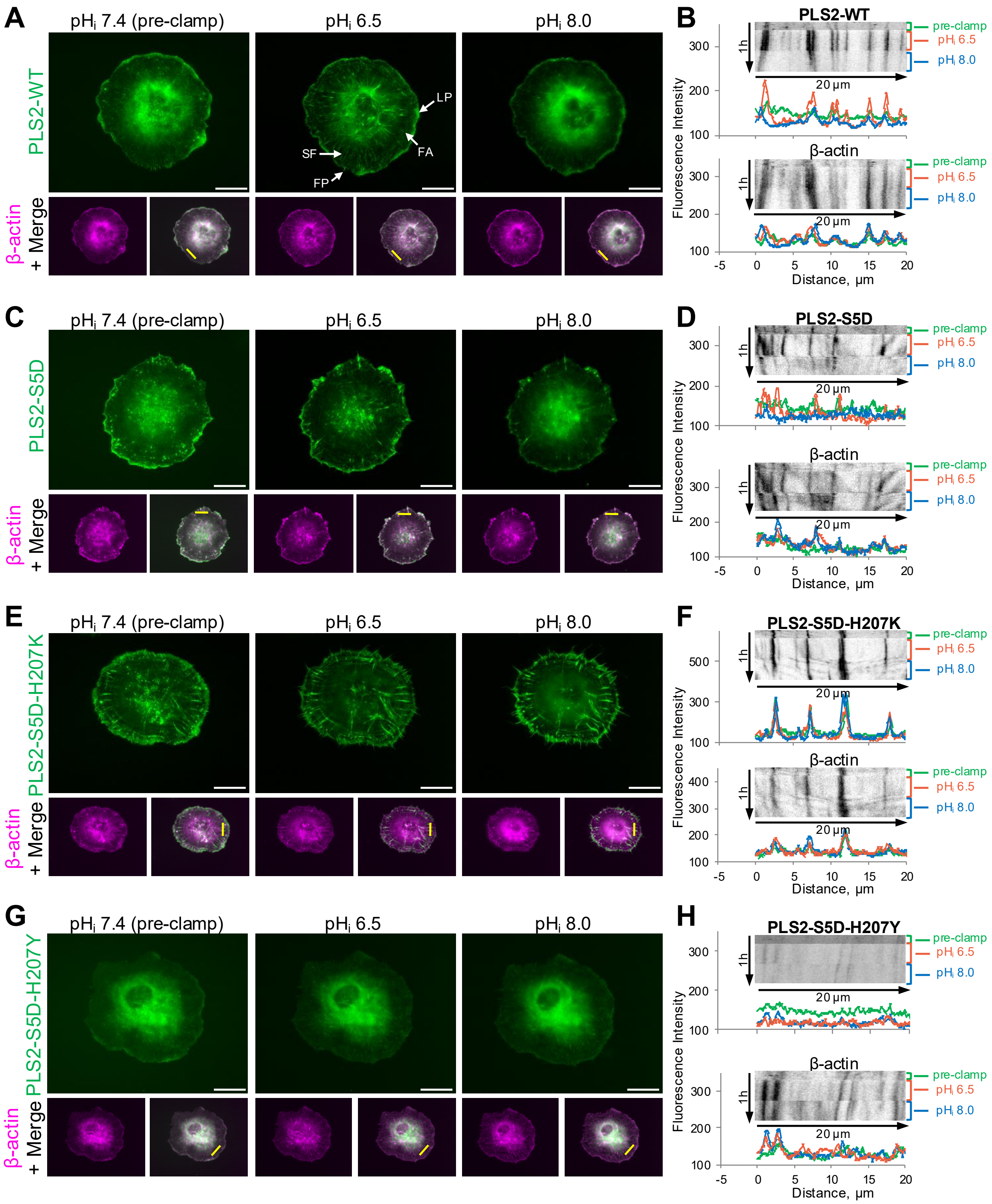

Figure S5. PLS2 shows pH-dependent cellular localization. (**A,C,E,F**) Time-lapse images of XTC cells co-transfected with mCherry-β-actin (magenta) and mEmerald-tagged WT (**A**), S5D (**C**), S5D/H207K (**E**), or S5D/H207Y (**F**) PLS2 constructs (green). Cells were imaged pre-clamped and sequentially clamped with nigericin buffers at pH 6.5 and 8.0. First frames of the time-lapse series at each pH_i condition (pre-clamping pH_i 7.4; pH_i 6.5; pH_i 8.0) are shown. Scale bars are 20 µm. LP, lamellipodia; FA, foacal adhesions; FP, filopodia; SF, stress fibers. (**B,D,F,H**) Kymographs and line plot profiles (taken from the regions indicated in the merge images in A, C, E, F by yellow lines, which also serve as 20 µm scale bars) are shown in two channels (for the corresponding PLS2 construct and β-actin). Plot profiles (*i.e.*, fluorescence intensity traces plotted versus distance indicated by the yellow lines in A, C, E, F at each pH_i condition) are color-coded for each pH_i according to the color scheme on the kymographs. See also Videos S6-S9.

Figure S6

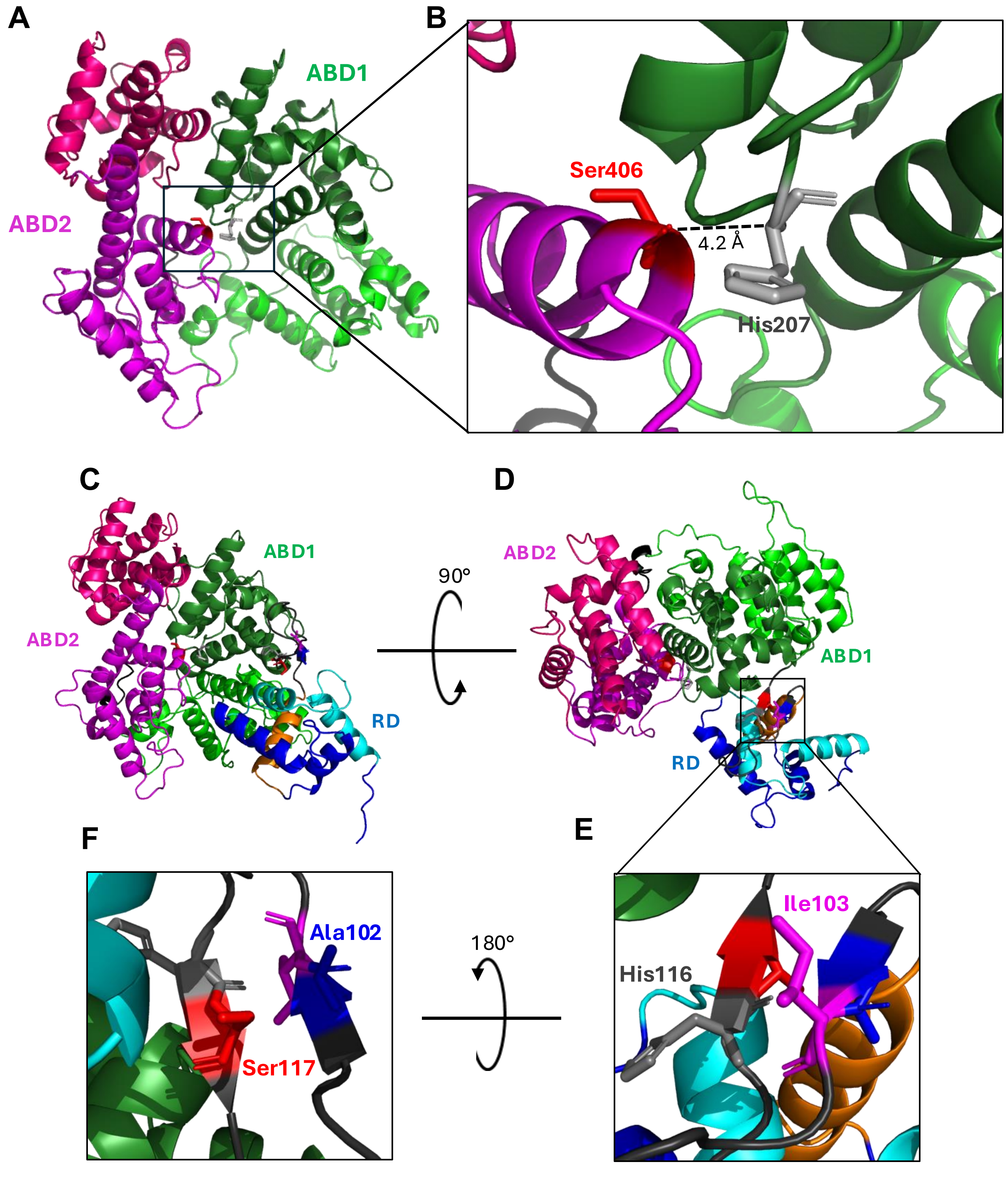

Figure S6. Relative location of selected residues in tertiary structure of PLS2 as predicted by AlphaFold2. (**A,B**) Actin-binding core of PLS2 (RD is not shown). Domains are indicated by color: dark green, CH1; green, CH2; magenta, CH3; pink, CH4. (B) is zoomed image of (A) to show location of Ser406 (red sticks) and His207 (grey sticks). Distance between the α-carbon atoms of Ser406 and His207 is indicated as black dashed line. (**C-F**) Full-length PLS2. Domains colored as in A, with additional domains indicated by color: light blue, EF-1; blue, EF-2; orange, CBM; dark grey, linker region between CBM and ABD1 (a.a. 97 to 120). Selected residues located in the linker between ABD1 and CBM are shown as sticks: Ser117 (red), His116 (grey), Ala102 (blue), and Ile103 (magenta). (D) Full-length PLS2 as in (C) rotated 90° about the y-axis. (E) A zoomed image of (D) to show relative location of His116 and its H-bonding partner Ile103 in an anti-parallel β-sheet. (F) image of (E) rotated 180° about the y-axis to show relative location of Ser117 and its H-bonding partner Ala102 in an antiparallel β-sheet. Notice that the RD position relative to the core is unlikely to be predicted correctly.

Figure S7

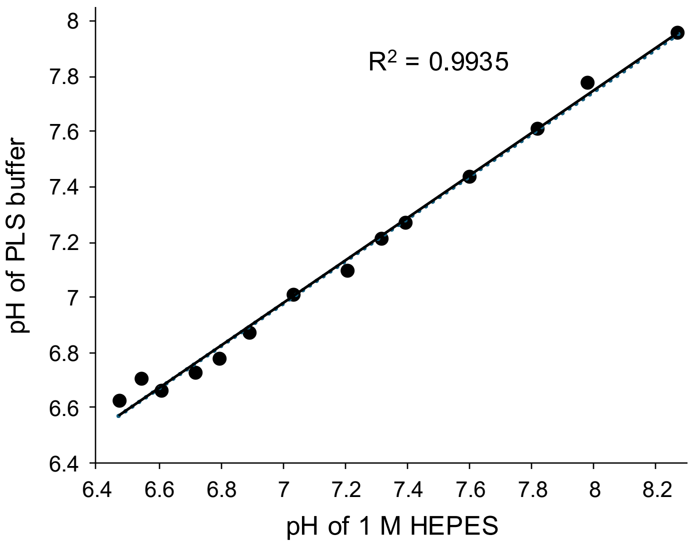

Figure S7. Standard curve for PLS buffer pH estimation. Final pH values of assembled PLS buffers are plotted against the pH of 1 M HEPES stock solution used to produce each of them. Dots represent data; line is a linear fit. Related to Methods (*Buffer pH determination* section).

Supplementary Tables

Table S1

Analysis of variance with Tukey's post-hoc test on the data from light scattering of F-actin in the presence of PLS2 with varying pH. Final light scattering intensity at 3000 s for each pH condition compared to each other pH condition; n.s. is non-significant.
Related to Figure 1D.

pH condition	Δ mean	SE of mean	q Value	Probability	Alpha	Significance	Lower control limit	Upper control limit
pH 6.8 vs pH 6.6	-35260.57719	170153.2098	0.29307	1	0.05	n.s.	-615701.5249	545180.3705
pH 7.0 vs pH 6.6	49031.26582	159163.7536	0.43566	0.99998	0.05	n.s.	-493921.5241	591984.0557
pH 7.0 vs pH 6.8	84291.84301	159163.7536	0.74896	0.99929	0.05	n.s.	-458660.9469	627244.6329
pH 7.2 vs pH 6.6	134771.1012	170153.2098	1.12014	0.99144	0.05	n.s.	-445669.8465	715212.0489
pH 7.2 vs pH 6.8	170031.6784	170153.2098	1.4132	0.96864	0.05	n.s.	-410409.2694	750472.6261
pH 7.2 vs pH 7.0	85739.83536	159163.7536	0.76182	0.99921	0.05	n.s.	-457212.9545	628692.6253
pH 7.4 vs pH 6.6	182549.8509	170153.2098	1.51725	0.95469	0.05	n.s.	-397891.0968	762990.7986
pH 7.4 vs pH 6.8	217810.4281	170153.2098	1.81031	0.89482	0.05	n.s.	-362630.5196	798251.3758
pH 7.4 vs pH 7.0	133518.5851	159163.7536	1.18635	0.98806	0.05	n.s.	-409434.2048	676471.375
pH 7.4 vs pH 7.2	47778.74975	170153.2098	0.39711	0.99999	0.05	n.s.	-532662.198	628219.6975
pH 7.6 vs pH 6.6	97086.77232	170153.2098	0.80693	0.99886	0.05	n.s.	-483354.1754	677527.72
pH 7.6 vs pH 6.8	132347.3495	170153.2098	1.09999	0.99231	0.05	n.s.	-448093.5982	712788.2972
pH 7.6 vs pH 7.0	48055.5065	159163.7536	0.42699	0.99998	0.05	n.s.	-494897.2834	591008.2964
pH 7.6 vs pH 7.2	-37684.32886	170153.2098	0.31321	1	0.05	n.s.	-618125.2766	542756.6189
pH 7.6 vs pH 7.4	-85463.07861	170153.2098	0.71032	0.9995	0.05	n.s.	-665904.0263	494977.8691
pH 7.8 vs pH 6.6	-108448.3259	170153.2098	0.90136	0.99771	0.05	n.s.	-688889.2737	471992.6218
pH 7.8 vs pH 6.8	-73187.74874	170153.2098	0.60829	0.99982	0.05	n.s.	-653628.6965	507253.199
pH 7.8 vs pH 7.0	-157479.5918	159163.7536	1.39925	0.97025	0.05	n.s.	-700432.3817	385473.1982
pH 7.8 vs pH 7.2	-243219.4271	170153.2098	2.0215	0.83244	0.05	n.s.	-823660.3748	337221.5206
pH 7.8 vs pH 7.4	-290998.1769	170153.2098	2.41861	0.6814	0.05	n.s.	-871439.1246	289442.7709
pH 7.8 vs pH 7.6	-205535.0983	170153.2098	1.70829	0.91924	0.05	n.s.	-785976.046	374905.8495
pH 8.0 vs pH 6.6	-554992.2464	159163.7536	4.93126	0.04308	0.05	significant	-1097950	-12039.4565
pH 8.0 vs pH 6.8	-519731.6692	159163.7536	4.61796	0.06638	0.05	n.s.	-1062680	23221.12069
pH 8.0 vs pH 7.0	-604023.5122	147357.0022	5.79693	0.01241	0.05	significant	-1106700	-101346.9061
pH 8.0 vs pH 7.2	-689763.3476	159163.7536	6.12874	0.00762	0.05	significant	-1232720	-146810.5577
pH 8.0 vs pH 7.4	-737542.0973	159163.7536	6.55326	0.00408	0.05	significant	-1280490	-194589.3074
pH 8.0 vs pH 7.6	-652079.0187	159163.7536	5.7939	0.01247	0.05	significant	-1195030	-109126.2288
pH 8.0 vs pH 7.8	-446543.9205	159163.7536	3.96767	0.15445	0.05	n.s.	-989496.7104	96408.86944

Table S2

Analysis of variance with Tukey's post-hoc test on the data from low-speed co-sedimentation of F-actin in the presence of PLS2 with varying pH. Fraction of F-actin pelleted using a low-speed co-sedimentation assay in each pH condition compared to each other pH condition; n.s. is non-significant.
Related to Figure 1D.

pH condition	Δ mean	SE of mean	q Value	Probability	Alpha	Significance	Lower control limit	Upper control limit
pH 6.8 vs pH 6.6	0.13089	0.07612	2.43173	0.67619	0.05	n.s.	-0.13266	0.39444
pH 7.0 vs pH 6.6	0.21019	0.07612	3.90494	0.1736	0.05	n.s.	-0.05336	0.47374
pH 7.0 vs pH 6.8	0.0793	0.07612	1.47321	0.96021	0.05	n.s.	-0.18425	0.34285
pH 7.2 vs pH 6.6	0.19703	0.07612	3.66048	0.2291	0.05	n.s.	-0.06652	0.46058
pH 7.2 vs pH 6.8	0.06614	0.07612	1.22874	0.98501	0.05	n.s.	-0.19741	0.32969
pH 7.2 vs pH 7.0	-0.01316	0.07612	0.24447	1	0.05	n.s.	-0.27671	0.25039
pH 7.4 vs pH 6.6	0.20498	0.07612	3.80801	0.19414	0.05	n.s.	-0.05857	0.46853
pH 7.4 vs pH 6.8	0.07408	0.07612	1.37628	0.97211	0.05	n.s.	-0.18947	0.33763
pH 7.4 vs pH 7.0	-0.00522	0.07612	0.09693	1	0.05	n.s.	-0.26877	0.25833
pH 7.4 vs pH 7.2	0.00794	0.07612	0.14754	1	0.05	n.s.	-0.25561	0.27149
pH 7.6 vs pH 6.6	0.20107	0.07612	3.73538	0.21078	0.05	n.s.	-0.06248	0.46462
pH 7.6 vs pH 6.8	0.07017	0.07612	1.30365	0.97919	0.05	n.s.	-0.19338	0.33372
pH 7.6 vs pH 7.0	-0.00913	0.07612	0.16956	1	0.05	n.s.	-0.27268	0.25442
pH 7.6 vs pH 7.2	0.00403	0.07612	0.07491	1	0.05	n.s.	-0.25952	0.26758
pH 7.6 vs pH 7.4	-0.00391	0.07612	0.07263	1	0.05	n.s.	-0.26746	0.25964
pH 7.8 vs pH 6.6	0.09868	0.07612	1.83323	0.88754	0.05	n.s.	-0.16487	0.36223
pH 7.8 vs pH 6.8	-0.03222	0.07612	0.5985	0.99983	0.05	n.s.	-0.29577	0.23133
pH 7.8 vs pH 7.0	-0.11151	0.07612	2.07171	0.81437	0.05	n.s.	-0.37506	0.15204
pH 7.8 vs pH 7.2	-0.09836	0.07612	1.82725	0.88913	0.05	n.s.	-0.36191	0.16519
pH 7.8 vs pH 7.4	-0.1063	0.07612	1.97478	0.84632	0.05	n.s.	-0.36985	0.15725
pH 7.8 vs pH 7.6	-0.10239	0.07612	1.90215	0.86832	0.05	n.s.	-0.36594	0.16116
pH 8.0 vs pH 6.6	-0.05992	0.07612	1.11311	0.9915	0.05	n.s.	-0.32347	0.20363
pH 8.0 vs pH 6.8	-0.19081	0.07612	3.54484	0.25975	0.05	n.s.	-0.45436	0.07274
pH 8.0 vs pH 7.0	-0.27011	0.07612	5.01805	0.04252	0.05	significant	-0.53366	-0.00656
pH 8.0 vs pH 7.2	-0.25695	0.07612	4.77359	0.05877	0.05	n.s.	-0.5205	0.0066
pH 8.0 vs pH 7.4	-0.26489	0.07612	4.92112	0.04838	0.05	significant	-0.52844	-0.00134
pH 8.0 vs pH 7.6	-0.26098	0.07612	4.84849	0.05326	0.05	n.s.	-0.52453	0.00257
pH 8.0 vs pH 7.8	-0.15859	0.07612	2.94634	0.46326	0.05	n.s.	-0.42214	0.10496

Table S3

Kinetic and equilibrium parameters for association and dissociation of FM-ABD2 to and from RD-ABD1 at varying buffer pH. k_{off} ($n \geq 4$) and K_d ($n = 3$) measured in equilibrium assays are reported as mean ± SD. Error for k_{on} represents curve fit error. K_d derived from kinetics was calculated using the k_{off} and k_{on} for each specified pH.
Related to Figure 3.

pH	k_{on}	k_{off}	K_d derived from kinetics (nM)	K_d from equilibrium (nM)
6.6	1.94 ± 0.02	0.010 ± 0.003	5.1	–
7.0	1.60 ± 0.02	0.009 ± 0.002	5.7	4.5 ± 0.3
7.4	1.24 ± 0.02	0.013 ± 0.002	11	–
7.8	1.07 ± 0.02	0.008 ± 0.001	7.7	–
8.0	1.010 ± 0.008	0.008 ± 0.001	7.6	8.7 ± 0.8

Table S4

Analysis of variance with Tukey's post-hoc test on the data from high-speed co-sedimentation experiments. n.s. is non-significant.
Related to Figure 4D.

pH condition	Δ mean	SE of mean	q Value	Probability	Alpha	Significance	Lower control limit	Upper control limit
ABD1 pH 7 vs. ABD1 pH 8	0.2106	0.03514	8.477	0.0007	0.05	significant	0.09260	0.3286
ABD1 pH 7 vs. ABD1 H207K pH 7	0.01124	0.03514	0.4525	0.9994	0.05	ns	-0.1068	0.1293
ABD1 pH 7 vs. ABD1 H207K pH 8	0.3031	0.03514	12.20	<0.0001	0.05	significant	0.1851	0.4211
ABD1 pH 7 vs. ABD1 H207Y pH 7	0.1379	0.03514	5.552	0.0192	0.05	significant	0.01992	0.2560
ABD1 pH 7 vs. ABD1 H207Y pH 8	0.2935	0.03514	11.81	<0.0001	0.05	significant	0.1754	0.4115
ABD1 pH 8 vs. ABD1 H207K pH 7	-0.1994	0.03514	8.025	0.0011	0.05	significant	-0.3174	-0.08136
ABD1 pH 8 vs. ABD1 H207K pH 8	0.09247	0.03514	3.722	0.1625	0.05	ns	-0.02555	0.2105
ABD1 pH 8 vs. ABD1 H207Y pH 7	-0.07268	0.03514	2.925	0.3627	0.05	ns	-0.1907	0.04534
ABD1 pH 8 vs. ABD1 H207Y pH 8	0.08285	0.03514	3.335	0.2444	0.05	ns	-0.03517	0.2009
ABD1 H207K pH 7 vs. ABD1 H207K pH 8	0.2918	0.03514	11.75	<0.0001	0.05	significant	0.1738	0.4099
ABD1 H207K pH 7 vs. ABD1 H207Y pH 7	0.1267	0.03514	5.099	0.0330	0.05	significant	0.008675	0.2447
ABD1 H207K pH 7 vs. ABD1 H207Y pH 8	0.2822	0.03514	11.36	<0.0001	0.05	significant	0.1642	0.4002
ABD1 H207K pH 8 vs. ABD1 H207Y pH 7	-0.1652	0.03514	6.647	0.0053	0.05	significant	-0.2832	-0.04713
ABD1 H207K pH 8 vs. ABD1 H207Y pH 8	-0.009622	0.03514	0.3873	0.9997	0.05	ns	-0.1276	0.1084
ABD1 H207Y pH 7 vs. ABD1 H207Y pH 8	0.1555	0.03514	6.260	0.0083	0.05	significant	0.03751	0.2735

Table S5

Kinetic parameters for association and dissociation of FM-ABD2 to and from RD-ABD1 variants at pH 7.0 and 8.0. k_{off} values reported as mean ± SD (n ≥ 4). Error for k_{on} represents curve fit error. K_d derived from kinetics was calculated using the k_{off} and k_{on} for the specified pH and construct. Since the dissociation rate of RD-ABD1-H207K at pH 8.0 followed a double exponential fit with 62% and 38% amplitudes (A), two corresponding k_{off} values are reported.
Related to Figure 4E,F.

		k_{on}	k_{off}	K_d derived from kinetics (nM)
RD-ABD1	pH 7.0	1.60 ± 0.02	0.009 ± 0.002	5.7
	pH 8.0	1.01 ± 0.01	0.008 ± 0.001	7.6
RD-ABD1-H207K	pH 7.0	1.59 ± 0.02	0.039 ± 0.001	24
	pH 8.0	1.12 ± 0.02	0.097 ± 0.004 (0.62 A)	87
			0.016 ± 0.002 (0.38 A)	14
RD-ABD1-H207Y	pH 7.0	1.87 ± 0.02	0.0021 ± 0.0001	1.1
	pH 8.0	1.21 ± 0.01	0.0026 ± 0.0004	2.2

Supplementary Video Captions

Videos S1-S5 are related to Figure 1G-I.

In Videos S1-S3, circles indicate pointed filament ends; arrowheads indicate growing barbed filament ends; arrows indicate the direction of filament elongation from the barbed ends (see Methods "*In vitro TIRF microscopy*" for details); individual colors distinguish individual filaments.

Video S1. An example of parallel bundles formed by PLS3 revealed by TIRF microscopy time-lapse imaging.

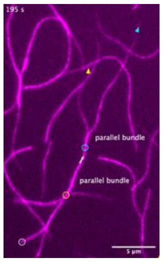

Video S2. An example of anti-parallel bundles formed by PLS3 revealed by TIRF microscopy time-lapse imaging.

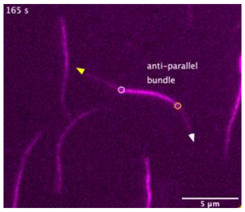

Video S3. An example of bundles with mixed polarity formed by PLS3 revealed by TIRF microscopy time-lapse imaging.

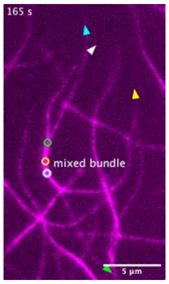

Video S4. Representative examples of bundles formed by PLS3 at pH 7.0 imaged by TIRF microscopy. Arrows indicate ends of a formed bundle; individual colors distinguish individual bundles.

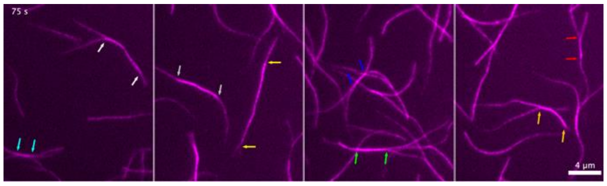

Video S5. Representative examples of bundles formed by PLS3 at pH 8.0 imaged by TIRF microscopy. Arrows indicate ends of a formed bundle; individual colors distinguish individual bundles. Note unzipping and filament rearrangement events (some are indicated by arrowheads).

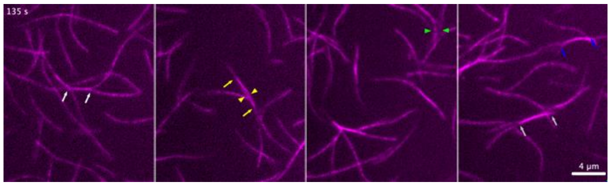

Videos S6-S9 are related to Figure S5.

Video S6. Time-lapse imaging of cells transiently co-transfected with mCherry-β-actin and mEmerald-PLS2-WT sequentially clamped at the indicated pH values.

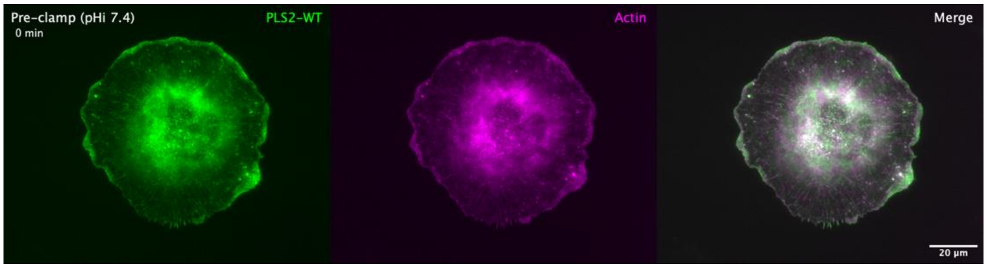

Video S7. Time-lapse imaging of cells transiently co-transfected with mCherry-β-actin and mEmerald-PLS2-S5D sequentially clamped at the indicated pH values.

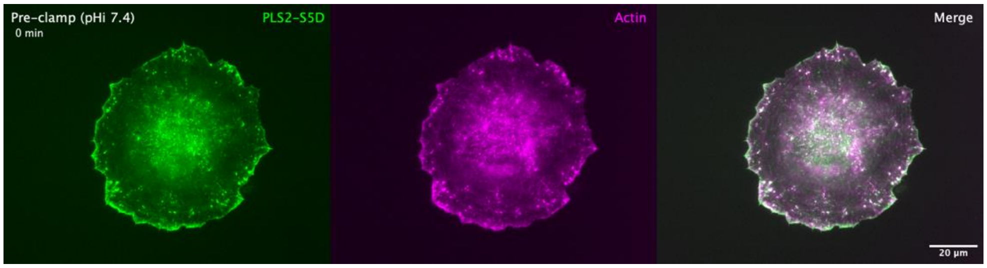

Video S8. Time-lapse imaging of cells transiently co-transfected with mCherry-β-actin and mEmerald-PLS2-S5D/H207K sequentially clamped at the indicated pH values.

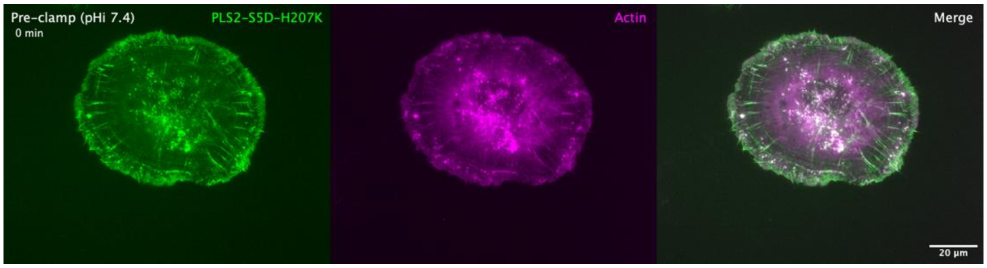

Video S9. Time-lapse imaging of cells transiently co-transfected with mCherry-β-actin and mEmerald-PLS2-S5D/H207Y sequentially clamped at the indicated pH values.

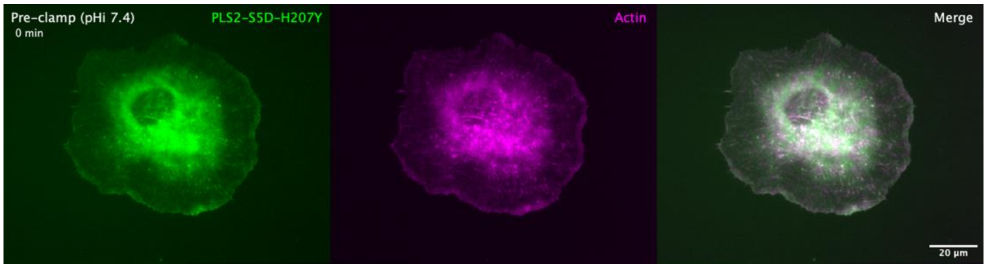

Supplement: Supplemental Material [file NIHMS2097282-supplement-Supplemental_Material.pdf]
