# Supplementary material for: Human Plastins are Novel Cytoskeletal pH Sensors with a Reduced F-actin Bundling Capacity at Basic pH
Source: J Mol Biol. Author manuscript; Available in PMC 2025 Jul 30. (PMC12309418; doi:10.1016/j.jmb.2025.169306)

Uncropped gels, related to Figure 1C

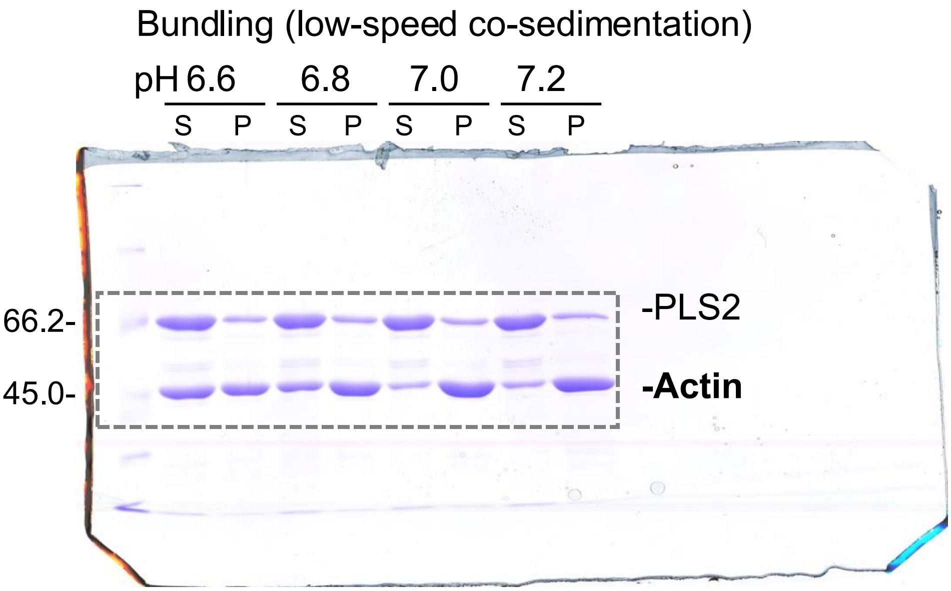

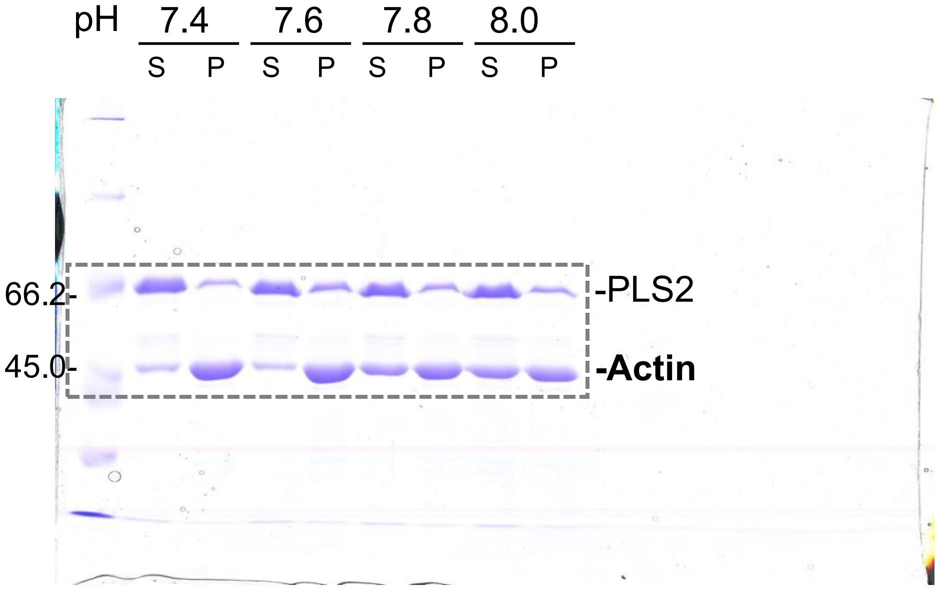

Uncropped gels, related to Figure 1E

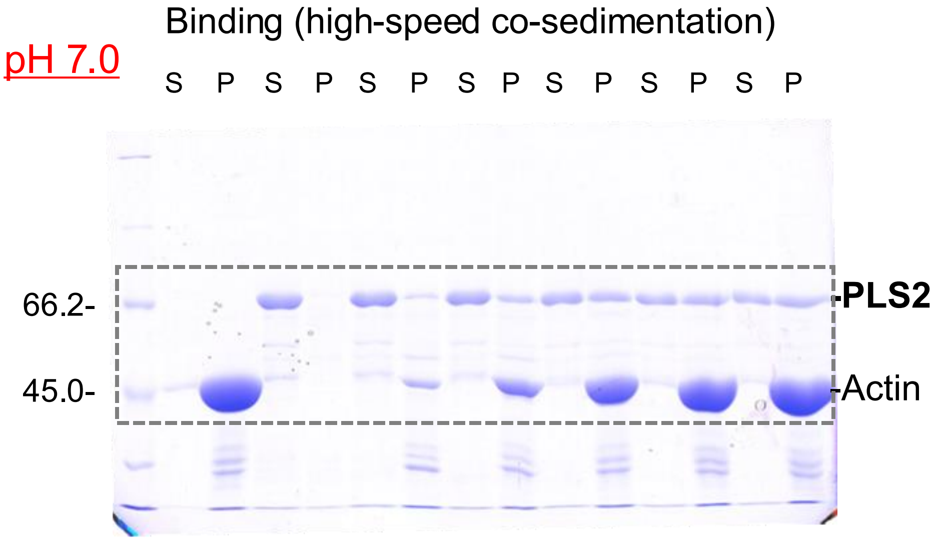

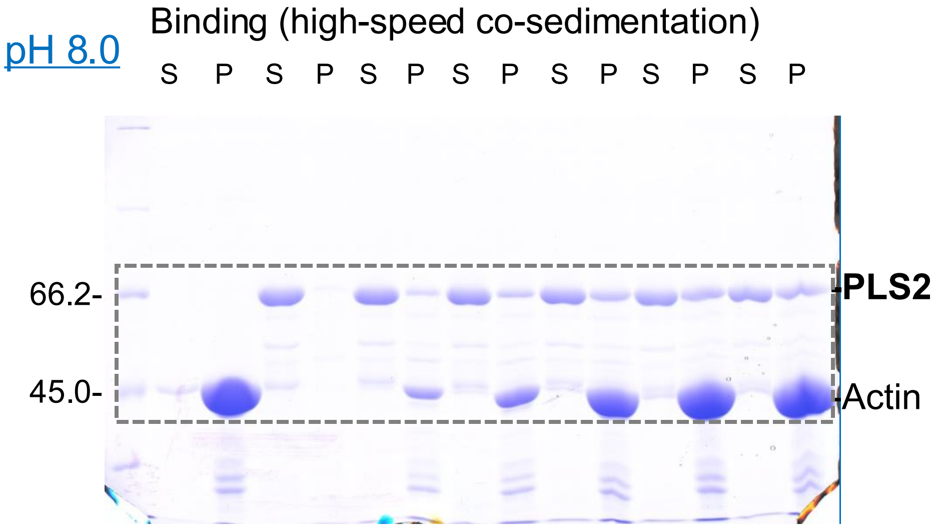

Uncropped gel, related to Figure 2B

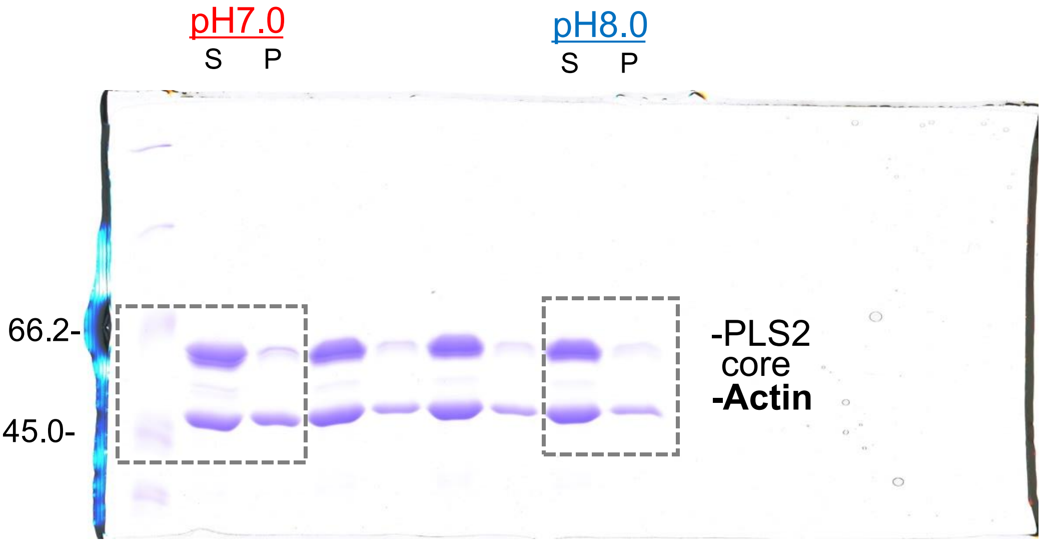

Uncropped gels, related to Figure 4B

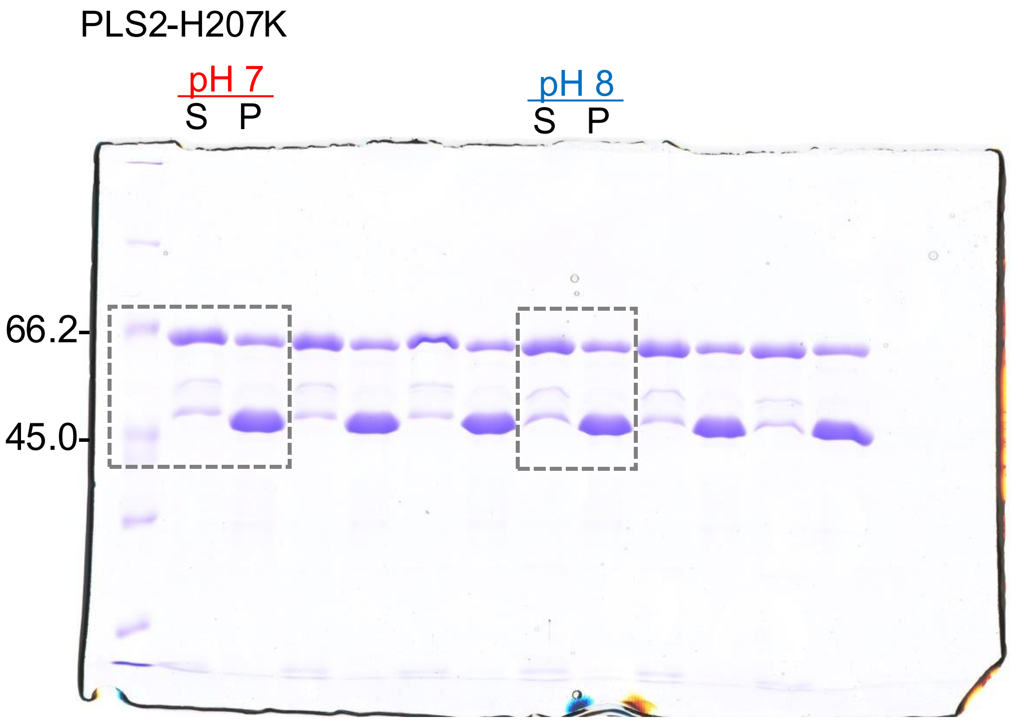

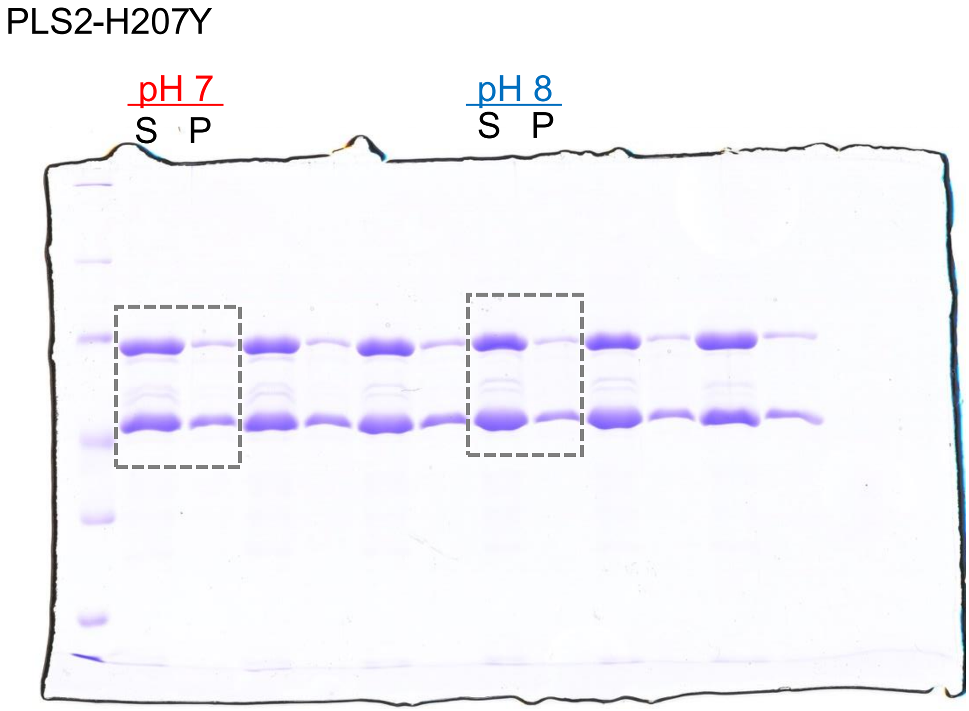

Uncropped gel, related to Figure S1C

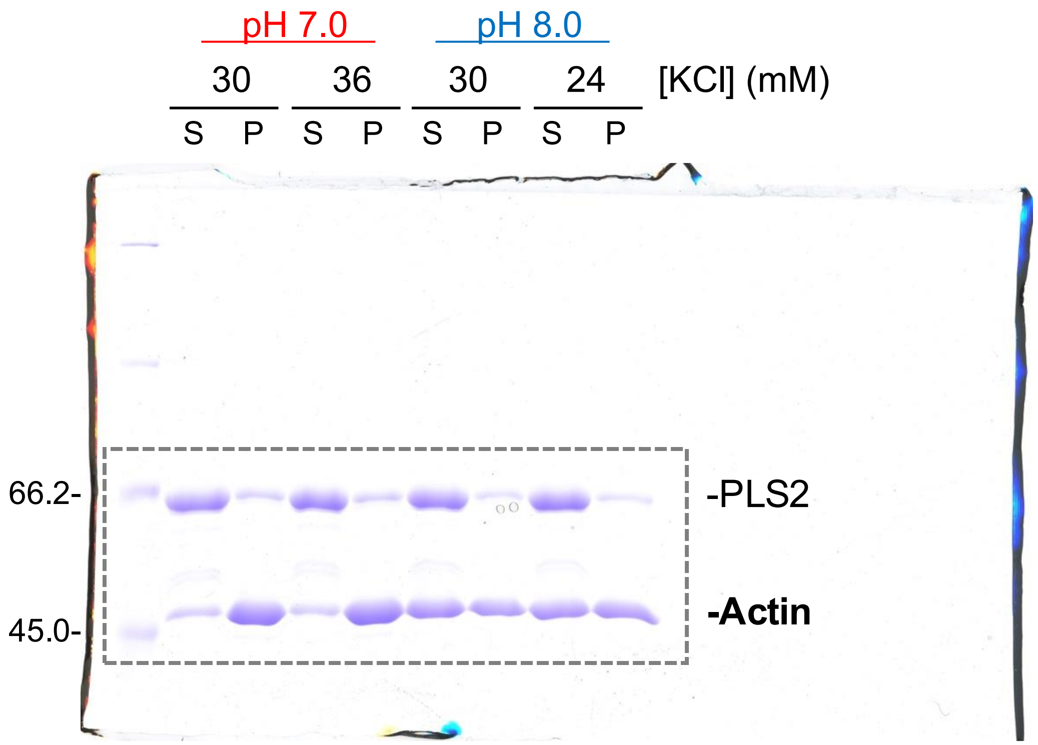

Uncropped gel, related to Figure S1F

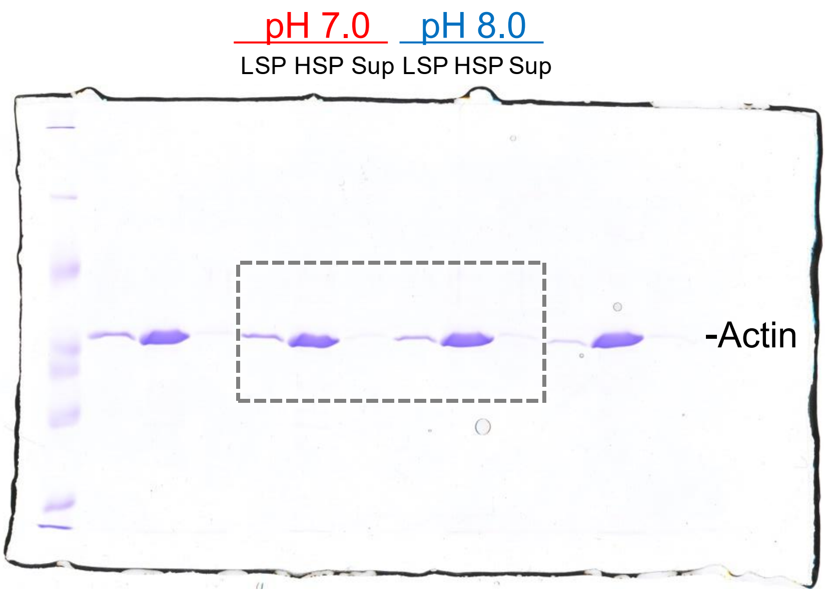

Supplement: Appendix-A1 [file NIHMS2097282-supplement-Appendix-A1.pdf]
